# Tissue architecture delineates field cancerization in BRAF^V600E^-induced tumor development

**DOI:** 10.1242/dmm.048887

**Published:** 2021-08-11

**Authors:** Elin Schoultz, Ellen Johansson, Carmen Moccia, Iva Jakubikova, Naveen Ravi, Shawn Liang, Therese Carlsson, Mikael Montelius, Konrad Patyra, Jukka Kero, Kajsa Paulsson, Henrik Fagman, Martin O. Bergo, Mikael Nilsson

**Affiliations:** 1 Sahlgrenska Center for Cancer Research, Department of Medical Chemistry and Cell Biology, Institute of Biomedicine, University of Gothenburg, SE-40530 Göteborg, Sweden; 2 Faculty of Medicine, Charles University, Hradec Kralove, Czech Republic; 3 Division of Clinical Genetics, Department of Laboratory Medicine, Lund University, Lund SE-22184, Sweden; 4 Department of Radiology, Institute of Clinical Sciences, University of Gothenburg, SE-41345 Göteborg, Sweden; 5 Department of Endocrinology, University of Turku, Åbo FI-20521, Finland; 6 Department of Clinical Pathology, Sahlgrenska University Hospital, Göteborg SE-41345, Sweden; 7 Department of Biosciences and Nutrition, Karolinska Institute, Huddinge SE-14183, Sweden

**Keywords:** Braf mutation, Cancer, Development, Oligoclonal, Oncogenic activation, Thyroid

## Abstract

Cancer cells hijack developmental growth mechanisms but whether tissue morphogenesis and architecture modify tumorigenesis is unknown. Here, we characterized a new mouse model of sporadic thyroid carcinogenesis based on inducible expression of BRAF carrying a Val600 Glu (V600E) point mutation (BRAFV600E) from the thyroglobulin promoter (*TgCreERT2*). Spontaneous activation of this *Braf*-mutant allele due to leaky activity of the Cre recombinase revealed that intrinsic properties of thyroid follicles determined BRAF-mutant cell fate. Papillary thyroid carcinomas developed multicentrically within a normal microenvironment. Each tumor originated from a single follicle that provided a confined space for growth of a distinct tumor phenotype. Lineage tracing revealed oligoclonal tumor development in infancy and early selection of BRAFV600E kinase inhibitor-resistant clones. Somatic mutations were few, non-recurrent and limited to advanced tumors. Female mice developed larger tumors than males, reproducing the gender difference of human thyroid cancer. These data indicate that BRAFV600E-induced tumorigenesis is spatiotemporally regulated depending on the maturity and heterogeneity of follicles. Moreover, thyroid tissue organization seems to determine whether a *BRAF*-mutant lineage becomes a cancerized lineage. The *TgCreERT2;BrafCA/+* sporadic thyroid cancer mouse model provides a new tool to evaluate drug therapy at different stages of tumor evolution.

## Abstract (precis)

**Summary:** This study investigates how a BRAF-mutant lineage becomes cancerized by escaping cell competition from non-mutant cells in a mouse model of sporadic thyroid cancer.

## INTRODUCTION

The cell of origin in differentiated thyroid cancer – the follicular cell – gives rise to two main tumor types: follicular thyroid carcinoma (FTC) and papillary thyroid carcinoma (PTC), both of which are overrepresented in women (Dralle et al., 2015; Williams, 2015). As indicated by the nomenclature, FTC and PTC possess different features of tumor growth and differentiation that influence clinical outcome; they are, therefore, considered as separate cancer entities (Dralle et al., 2015). The fact that mutation of different members of the small GTPase family RAS – mainly of *NRAS* and *BRAF –* predominantly associate with FTC and PTC, respectively (Howell et al., 2013; Xing, 2005), suggests that mutation identity can influence the morphogenesis of a distinct carcinoma phenotype. However, these driver mutations occur, although with varying frequencies, in nearly all thyroid cancer types. For example, PTC induced by BRAF carrying a Val600 to Glu (V600E) point mutation (BRAF^V600E^) comprises several subtypes including the follicular variant (Afkhami et al., 2016). A single PTC tumor may also display a mixture of growth patterns, the predominant one being decisive for diagnostic subtyping. Most PTCs have a low somatic mutation burden, indicating that genomic instability is not a critical factor except in advanced tumor stages (Cancer Genome Atlas Research Network, 2014). Although transcriptional profiling distinguishes between PTCs as being ‘*RAS*-like’ and ‘*BRAF*-like’ neoplasms with different levels of tumor dedifferentiation and aggressiveness (Cancer Genome Atlas Research Network, 2014), the underlying mechanisms of the morphogenetic traits that give rise to heterogeneous tumor phenotypes in the thyroid are unknown.

Tumor heterogeneity conceptually implies diversification of cancer cell properties – genetically, morphologically and biochemically – that are involved in tumor progression. According to the clonal evolution model (Swanton, 2012), tumors arise from a single mutated cell that, upon accumulation of additional somatic mutations, gives rise to a tumor clone that possesses a growth advantage. Further branched evolution of heterogeneous subclones contributes to tumor progression and provides a selection mechanism to escape anti-cancer drug treatment (Turajlic et al., 2019). An alternative, less-recognized mechanism concerns the possible involvement of a multiclonal tumor origin (Parsons, 2018), which implies that two or more independent clones cooperate in tumor development and heterogeneous tumor growth. Such clonal cooperation might be necessary for mutant cells to resist competition with non-mutant cells. Escaping surveillance mechanisms associated with normal tissue homeostasis (Bowling et al., 2019) may not only be decisive for tumor development but also influence later stages of cancer progression (Cheung et al., 2016; Echeverria et al., 2018; Reeves et al., 2018). In thyroid cancer patients, mutation analysis infers that PTC, the most common type of thyroid cancer, is a strictly monoclonal tumor (Cancer Genome Atlas Research Network, 2014). However, as recently reviewed (Fugazzola et al., 2020), there are several divergent reports of genetically heterogeneous tumor cell populations that argue against the prevailing concept of monoclonality, suggesting that PTC development comprises subclonal or even oligoclonal events. To date, there are no experimental studies that address the clonal origin of differentiated thyroid cancer and the role of clonality in thyroid tumor development.

Mouse models have been invaluable in studies of thyroid cancer progression and the evaluation of targeted therapies (Landa and Knauf, 2019). However, since conditional expression of oncogenes, such as mutant BRAF, encompasses the majority of cells, there are no current models that reliably replicate tumor initiation and early events of the carcinogenic process within a preserved thyroid tissue microenvironment. Notably, loss of thyroid function, accompanying synchronous activation of *Braf^CA^* that encodes the BRAF^V600E^ oncoprotein, rapidly generates pronounced goitre growth and leads to global disruption of the normal follicular organization (Chakravarty et al., 2011), which invalidates the monitoring of any discrete cellular changes presumed to characterize focal tumorigenesis. To overcome these obstacles, we have adopted a novel tumorigenic approach based on spontaneous Cre-mediated recombination, which occurs at significant levels under non-induced conditions in *TgCreER^T2^;Braf^CA/+^* mice that conditionally express Braf^V600E^ within the thyroid (Charles et al., 2011). This enabled us to investigate through lineage tracing the earliest stages of Braf^V600E^-induced tumor development and to elucidate the clonal origin of tumor heterogeneity. Our results indicate that tissue organization designated by follicle heterogeneity delimits the effective cancerization field and, hence, determines the fate of thyroid cells subjected to oncogene activation. Postnatal nascent follicles are particularly susceptible to *Braf* mutations, as they are prone to develop oligoclonal lesions that escape competition and give rise to PTCs of diverse phenotypes in mice.

## RESULTS

### Spontaneous Braf^CA^ activation in mouse thyroid generates multifocal heterogeneous PTC tumors with accelerated growth in females

**Fig. 1.**
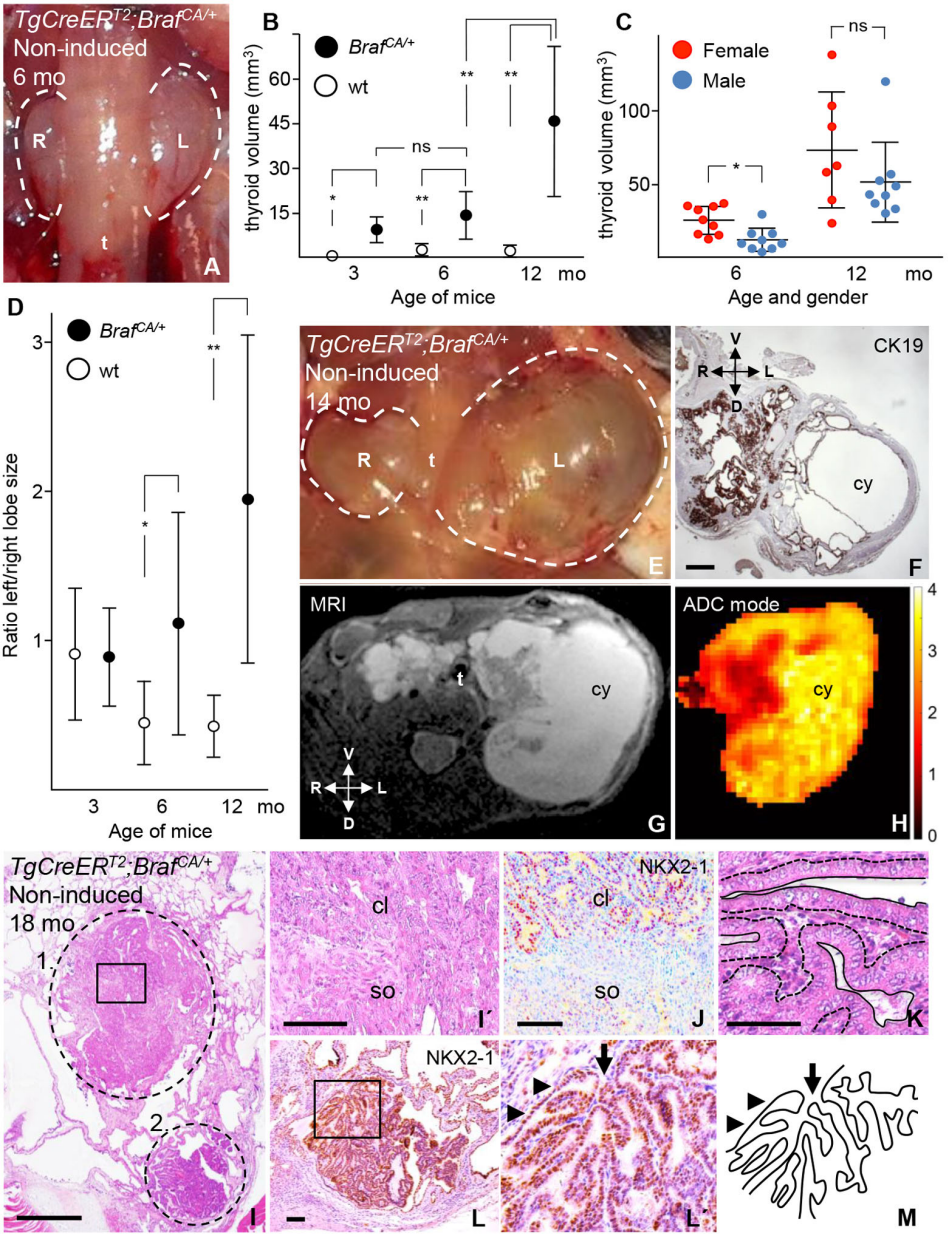
**Occurrence of papillary thyroid carcinoma (PTC) in non-induced *TgCreER^T2^;Braf^CA/+^* mice devoid of tamoxifen injections.** Data are from wild-type (wt) and *Braf^CA^* mutant mice at age 3-18 months (mo). Thyroid volumes were estimated from lobe diameter measurements. (A,E) Shown are *in situ* micrographs of frontal views of enlarged thyroids. (B) Thyroid volumes plotted over time. (C) Thyroid volume in relation to the sex plotted over time. (D) Ratio of left to right lobe – i.e. asymmetric lobe growth – plotted over time. Mean±s.d; *P<0.005; **P<0.0001. For B and D, numbers of mice were wt (*n*=12) and mutant (*n*=17) at 3 months; wt (*n*=16) and mutants (*n*=20) at 6 months; wt (*n*=12) and mutants (*n*=18) at 12 months. (F) Immunostaining for cytokeratin 19 (CK19) showing increased protein levels, consistent with raised CK19 levels observed in human PTCs. (G,H) T2-weighted MRI image (G) of the same thyroid specimen as shown in E and F (for entire stack series, see Movie 1) and apparent diffusion coefficient (ADC) color map (H) of the same image. The color bar relates to solid (red) and cystic (yellow) tumors based on the ADC (µm^2^/ms). cy, corresponding cystic tumor portions. Used technology delimited resolution of images. (I-K) H&E staining showing inter- and intra-tumor heterogeneity of multifocal PTCs. Images of additional tumors present in the same thyroid specimen are shown in Figs S1 and S2. Two adjacent PTC tumor foci are encircled (1 and 2) and shown in I; the boxed area in circle 1 is shown magnified in I′, indicating a transition of the tumor growth pattern. Immunostaining for NKX2-1 indicating its downregulation in the solid tumor portion, is shown in J; the parallel section of J is shown in panel l′. Nuclear characteristics of tumor cells are shown in K – magnification of a region within the first PTC tumor (circle 1) from I; the interface between lumen and stroma of the tumor tissue is indicted by dashed lines. Immunostaining for NKX2-1 (L) within the second PTC tumor shown in I (circle 2). The boxed area in L is shown magnified in L′. (M) Sketch outlining the papillary tumor growth as shown in L′. Anatomical orientation: D, dorsal; L, left; R, right; V, ventral. cl, classic variant of PTC; so, solid variant of PTC; t, trachea. Arrows indicate the tumor stalk; arrowheads indicate the follicular wall enclosing the tumor. Scale bars: 500 µm (F,I), 100 µm (I′,J,L), 50 µm (K).

We obtained our sporadic thyroid cancer mouse model *TgCreER^T2^;Braf^CA/+^* by crossing the established *Braf^CA^* and *TgCreER^T2^* mouse lines (Dankort et al., 2007; Undeutsch et al., 2014) to conditionally express BRAF^V600E^ in the thyroid under control of the *thyroglobulin* (*Tg*) promoter, as previously reported (Charles et al., 2011). To yield our current *TgCreER^T2^;Braf^CA/+^* models, mutant mice were not induced by tamoxifen, in order to elucidate whether spontaneous activation of mutant BRAF due to leaky activity of the bacteriophagal Cre recombinase reproduce sporadic thyroid cancer development. *In situ* gland volume measurements showed that the thyroid gradually increased in size, with growth accelerating between 6 and 12 months of age in mutant animals (Fig. 1A,B). Notably, at 6 months, i.e. before great variations in thyroid size became evident, the gland was significantly larger in females than in male mutants (Fig. 1C). Consistent with the stochastic generation of tumors with different growth properties, the relative enlargement of the left and right thyroid lobes differed increasingly with age in the majority of mice (Fig. 1D and E). Interestingly, 83% of 12-months-old mutants (*n*=18) showed a higher left-to-right lobe ratio (Fig. 1D). The fact that larger tumors were predominant in the left lobe was reinforced by a trend of the opposite-lobe asymmetry in aging control animals (Fig. 1D). A single-sided preference is evident for thyroid developmental defects in both mice (Manley and Capecchi, 1995) and humans (Shabana et al., 2000), but has not previously been reported for neoplastic lesions. As documented by magnetic resonance imaging (MRI), heterogeneous tumor growth explained the lobe size differences (Fig. 1G,H; Movie 1). Mouse MRI also revealed that cystic tumors were predominantly located at the periphery of the lobes, whereas more solid tumor portions often took a central or medial location in the lobes.

Advanced tumor stages were further analyzed in serial-sectioned thyroids from non-induced *TgCreER^T2^;Braf^CA/+^* mice between 12 and 22 months (*n*=15). Consistent with PTC features in humans (Rorive et al., 2002), we observed increased levels of cytokeratin 19 (KRT19, hereafter referred to as CK19) in tumor cells of mutant mice (Fig. 1F; Fig. S1A,B). Moreover, reminiscent of PTC subtypes classified histologically, the tumors showed a highly variable growth pattern comprising (i) classic or conventional PTC with an abundance of papillary formations (Fig. 1I-M; Fig. S1B,C) and the characteristic ground glass nuclear features of the tumor cells (Fig. 1K); (ii) cystic PTC with hobnail-like features of the epithelial lining (Fig. S1E); (iii) solid variant PTC essentially devoid of papillary growth (Figs. S1G, S2A-A′) and; (iv) tall-cell variant PTC with an unusual cylindrical tumor cell shape (Fig. S2D,D′). Altered expression levels of the key thyroid transcription factor NKX2-1 (Fernández et al., 2015) were evident among tumors and also within a single tumor (Fig. 1J,L,L′; Fig. S2B,B′), presumably reflecting heterogeneous tumor properties. Notably, tumor cells invaded stroma-rich tissue either by collective migration with a preserved ability to form follicles (Figs S1D, S2D,D′) or by undergoing partial epithelial-mesenchymal transition (EMT) characterized by diminished expression and disrupted localization of E-cadherin (Figs S1G, S2B,B′ and S2C,D). Occasionally, tumor cells infiltrated extra-thyroidal tissues (Fig. S2D,D′).

Altogether, these findings indicated that the sporadic activation of *Braf^CA^* in mice generates multifocal thyroid carcinomas with distinctive growth patterns that mimic PTC subtypes in humans. Notably, the penetrance of *Braf* mutation was 100%, although with highly variable tumor sizes and phenotypes occurring among individuals as well as within the same gland, emphasizing the stochastic nature of thyroid tumorigenesis in this model. The development of larger tumors as seen in female mice is consistent with the well-known sex differences in the occurrence of PTC (Derwahl and Nicula, 2014; Dralle et al., 2015; Rahbari et al., 2010).

### BRAF^V600E^-induced thyroid tumorigenesis does not require additional protein-coding gene mutations

To elucidate the possible involvement of somatic mutations, tumor samples from *TgCreER^T2^;Braf^CA/+^* mice (*n*=5) between 4 and 12 months of age were subjected to whole-exome sequencing (WES). The entire lobe (age 4 months) or the most-tumorous part of the lobe (age 6-12 months) was excised and sequenced; one tamoxifen-treated mutant mouse (age 4 months, induced at weaning) was included for comparison. No mutations other than *BRAF^V600E^* were detected in tumors at 4 months, and few additional coding mutations (one to three per sample) accumulated between 6 and 12 months (Table 1; for quality data of sequencing, see Table S1). The mutational allele fraction varied between 0.05-0.14. None of the identified mutations reappeared, and none was previously reported in thyroid cancer, with the exception of *Pclaf* (Mizutani et al., 2005). Samples subjected to WES analysis were not morphologically examined but, since neoplastic cells predominated in most investigated tumorous tissues, the low allelic fraction of the identified mutations strongly suggest that all were subclonal. These data conform to previous reports indicating that most sporadic PTCs exhibit few genetic abnormalities apart from the oncogenic driver mutation (Cancer Genome Atlas Research Network, 2014). In the current model, it is thus evident that early tumor development, driven by mutant BRAF, does not require any additional mutations in protein-coding genes.

**Table 1.**
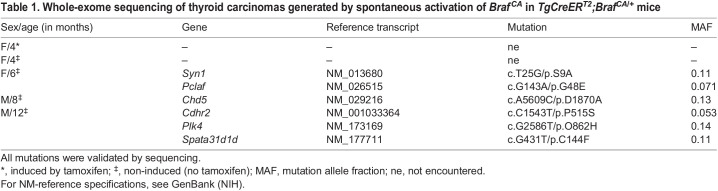
Whole-exome sequencing of thyroid carcinomas generated by spontaneous activation of Braf^CA^ in *TgCreER^T2^;Braf^CA/+^* mice

### Follicular neoplasms with different sensitivity to BRAF^V600E^ kinase inhibition develop within a structurally and functionally preserved thyroid microenvironment

**Fig. 2.**
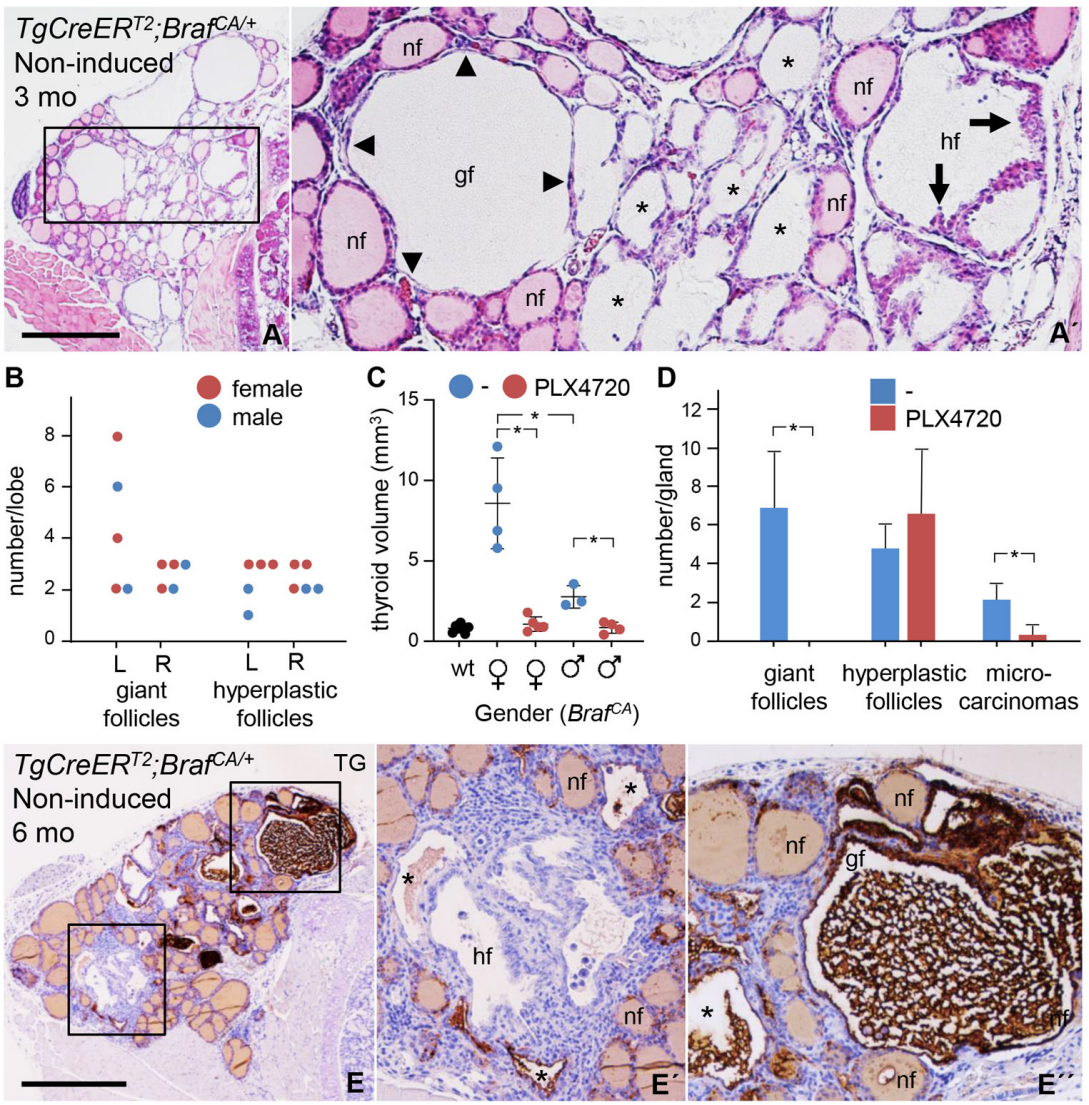
**Early stages of thyroid tumor development following spontaneous *Braf^CA^* activation in *TgCreER^T2^;Braf^CA/+^* mutant mice.** (A) Histogram showing H&E staining of heterotypic follicular abnormalities of the thyroid from a mouse aged 3 months (mo). The boxed area is shown magnified in A′. Asterisks indicate follicles with translucent interior, i.e. lack of colloid. (B-D) Quantitative assessment of changes within the thyroid in response to mutant BRAF kinase inhibition. Dietary pellets with PLX4720 were supplied daily at 417 ppm from 4 weeks onwards and until mice were killed aged 3 months; data were obtained from serial sections. The number of neoplastic follicles in untreated mutants (individual data) is plotted in B. Inhibition of thyroid enlargement (individual data; mean±s.d.; *P<0.005) is plotted in C. Heterogeneous drug response in neoplastic lesions. The mean±s.d. (*P<0.005) of (*n*): wt (5); untreated mutants (7), drug-treated mutants (9) is plotted in D. (E) Representative image of the thyroid from a mutant mouse aged 6 months, immunostained for thyroglobulin (TG), showing loss of TG in neoplastic lesions. Boxed areas in E are shown magnified in E′ and E′′. Asterisks in E′ and E′′ indicate follicles with altered shape and retained TG in the lumen. L, left lobe; R, right lobe. nf, normal follicle; hf, hyperplastic follicle; gf, giant follicle; arrows indicate hyperplastic epithelium; arrowheads indicate flat epithelium; wt, control wild-type (non-mutant) mice. Scale bars: 500 µm.

To understand the basis of heterogeneous tumor growth, earlier stages of tumor development in non-induced *TgCreER^T2^;Braf^CA/+^* mice were studied. At 3 months of age, mutant thyroids displayed a limited number of abnormal follicles surrounded by follicles of normal size and shape (Fig. 2A). The enlarged follicles were of two main types: a cell-rich type comprising a thickened and irregularly shaped epithelium, and a hollow type consisting of flattened cells and a distended epithelial lining (Fig. 2A′), hereafter referred to, respectively, as hyperplastic and dilated – including ‘giant’ variant – follicles. On average, each lobe contained two to three clusters of either type of follicular abnormality (Fig. 2B; *n*=10, serial-sectioned thyroids). Hyperplastic follicles were regularly embedded in the lobe interior, whereas giant follicles had a mostly peripheral location. The neoplastic nature of these early tissue alterations was confirmed by blocking mutant BRAF kinase activity with PLX4720, the precursor compound of PLX4032 or vemurafenib (for **V**600**E mu**tated B**RAF inhib**ition; we use this acronym synonymously to underline the principal similarity of PLX4720 and PLX4032), administered from the date of weaning, which virtually abolished thyroid enlargement in 3-months-old mice (Fig. 2C). Female and male thyroids showed equal numbers of hyperplastic and dilated follicles, and responded similarly to vemurafenib, although the relative size reduction was more pronounced in females due to the larger glands that develop in untreated mutants (Fig. 2B and C). Notably, vemurafenib inhibited giant follicle formation but did not fully prevent the generation of hyperplastic follicles and microcarcinomas distinguished by a papillary growth pattern (Fig. 2D; see below for further information of the papillary phenotype).

Mutant thyroids also consisted of normal-sized and moderately enlarged follicles with lumens conspicuously devoid of colloid (Fig. 2A′). A similar effect was evident globally in tamoxifen-induced mice before the compensatory high serum levels of thyroid stimulating hormone (TSH) stimulated goitrous growth (Fig. S3A-C). By contrast, confirming previous notions (Charles et al., 2011), non-induced *TgCreER^T2^;Braf^CA/+^* mice maintained systemic thyroid hormone homeostasis (Fig. S3C-E). This indicated that the resolution of colloid is likely to be TSH-independent and a direct effect of constitutive mitogen-activated protein kinase (MAPK) signaling in BRAF-mutant thyroid cells.

In mice, thyroglobulin (TG), the thyroid prohormone and main constituent of follicular colloid, is consistently downregulated in BRAF^V600E^-driven thyroid tumors, featuring a poorly differentiated state (Chakravarty et al., 2011). By using qPCR (Table S2), we confirmed that induced *Braf^CA^* activation virtually abolished thyroid-specific gene expression and diminished *Pax8*, a main transcriptional regulator of thyroid differentiation (Table S2) (Fernández et al., 2015). By contrast, under non-induced conditions, consistent with fewer numbers of oncogene-activated cells, transcription levels of *Pax8*, *Tg*, thyroid peroxidase (*Tpo*) and TSH receptor (*Tshr*) were partially reduced (Table S2). Morphologically, this corresponded to a markedly heterogeneous tissue consisting of normal follicles with homogeneous TG staining of the colloid, dilated follicles showing retention of agglutinated TG in the lumen and hyperplastic follicles that lack TG immunoreactivity (Fig. 2E and E′). Since microcarcinomas and manifest PTCs were also entirely TG negative (Fig. S4A,B) – confirming loss of differentiation of BRAF-mutant cells – these findings suggested that abnormal follicles with retained TG expression consist of both mutant and normal cells, and do not develop into tumors. Surprisingly, *Slc5a5*, encoding the sodium-iodide symporter (NIS), was equally suppressed after spontaneous and induced activation of *Braf^CA^* (Table S2). It is difficult to explain this other than by NIS expression being more broadly inhibited and encompassing not only BRAF-mutant cells. A bystander effect specifically affecting *Slc5a5* might occur, related to the fact that NIS is regulated independently and more sensitive to MAPK activation than other thyroid-specific genes (Chakravarty et al., 2011; Ingeson-Carlsson and Nilsson, 2013).

These findings indicated that mouse thyroid cells respond differently to sporadic activation of *Braf^CA^* and give rise to a heterogeneous population of follicular lesions of which only a fraction is tumorigenic. This tissue pattern of neoplastic growth, in fact, reminds of the generation of multinodular goiter that also occurs on the basis of normal follicle heterogeneity related to inherent variations in proliferating capacity of thyroid epithelial cells (Studer et al., 1989).

### BRAF^V600E^-induced loss of differentiation entails abolished Cre expression in mutant thyroid cells

**Fig. 3.**
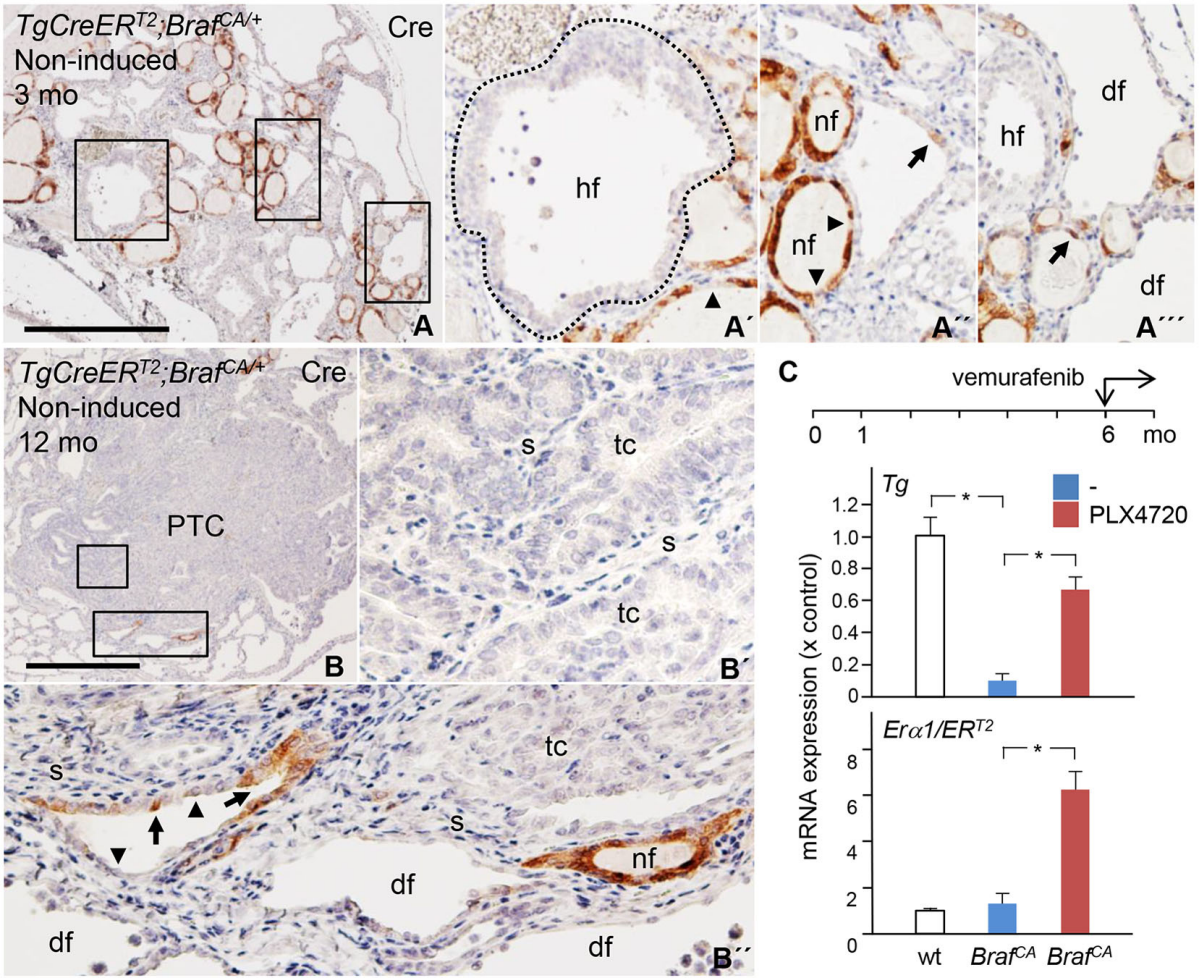
**Diminished *Cre* driver expression within thyroids of *TgCreER^T2^;Braf^CA/+^* mutant mice.** Data were obtained from mutant mice at age 3-12 months (mo) not treated with tamoxifen. (A,B) Tissue distribution of Cre-positive and Cre-negative cells (IHC staining). Boxed areas in A and B are shown magnified in A′-A′′′ and B′-B′′, respectively. (C) Recovery of *TgCreERT2* expression in mice (qPCR data) treated with PLX4720 (417 ppm; dietary pellets) at 6 months for the duration of 1 month. (Top) *Thyroglobulin* (*Tg*) transcript levels. (Bottom) Transcript levels of *Era1* (in wild type) and *ER*^*T2*^ (in mutants). nf, normal follicles; hf, hyperplastic follicles (encircled in A′); df, dilated follicles; PTC, papillary thyroid carcinoma; tc, tumor cells; s, stroma; arrows, Cre-positive cells; arrowheads, Cre-negative cells. Scale bars: 500 µm.

The next set of experiments was undertaken to elucidate in more detail how the pre-existing thyroid tissue organization influenced BRAF^V600E^-driven tumorigenesis in *TgCreER^T2^;Braf^CA/+^* mice. Owing to high background staining, including ubiquitous staining of nuclei (data not shown), immunohistochemistry (IHC) using antibodies against human BRAF^V600E^ oncoprotein was not feasible to localize and estimate the number of mutant cells. Therefore, we investigated whether Cre recombinase – whose expression correlates with that of TG – can be used as a marker to distinguish normal from BRAF-mutant cells. Indeed, confirming this relationship, only follicles of normal size and colloid structure consisted predominantly of Cre-positive cells, whereas hyperplastic follicles and tumors were devoid of Cre immunoreactivity (Fig. 3A-B′). Moreover, dilated follicles generally were Cre negative, with only few cells weakly stained for Cre (Fig. 3A″). It is noteworthy that seemingly normal follicles frequently consisted of both Cre-positive and Cre-negative epithelial cells (Fig. 3A″). As the expression levels of TG normally vary between thyroid follicles and even between adjacent follicular cells (Sellitti and Suzuki, 2014), it is possible that cells lacking Cre expression also comprise non-mutant cells. However, the presence of numerous and largely Cre-negative follicles devoid of colloid in peri-tumorous tissue (Fig. 3B,B″) suggests that mutant cells predominated in these enlarged follicles. Coordinated loss of *Tg* and *CreER^T2^* mRNA expression and rescue of *Tg* promoter activity by vemurafenib was confirmed in thyroid samples from non-induced *Braf^CA^* mice by using qPCR (Fig. 3C).

Together, these observations indicated that, within the BRAF-mutant thyroid cell population, the loss of expression of differentiation markers is accompanied by downregulation of Cre not only in manifest tumors and neoplastic follicles but also when present in follicles with more subtle alterations. As evidenced below, this enabled a tracing strategy to distinguish clonal fates of mutant thyroid cells with different temporal onset of spontaneous *Braf^CA^* activation.

### Tumor initiation starts perinatally, with oligoclonal growth of BRAF-mutant cells delineated by the originating follicle territory

**Fig. 4.**
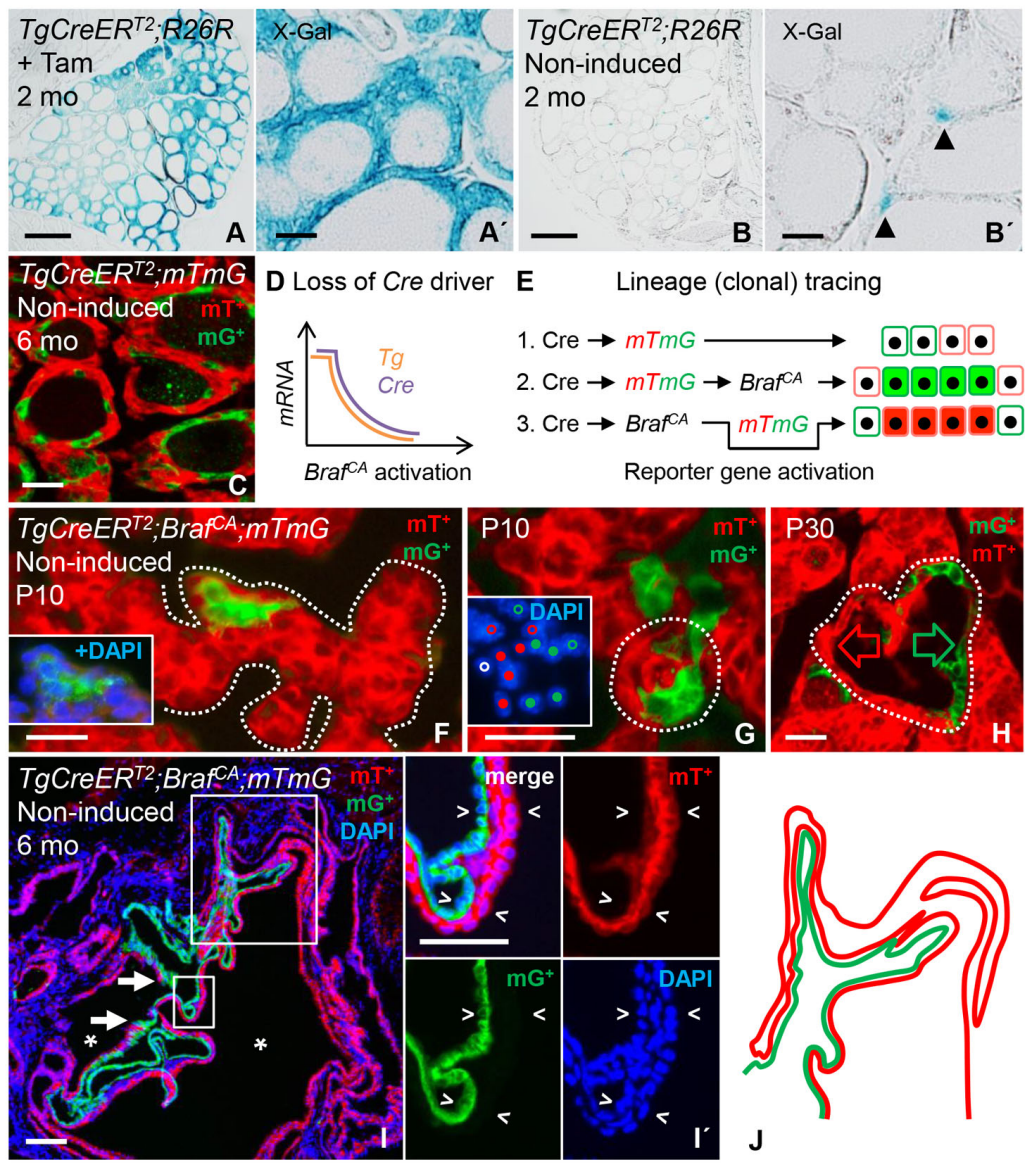
**Clonal tracing of mutant thyroid cells after spontaneous *Braf^CA^* activation.** (A,B) X-gal staining of thyroid cells from *TgCreER^T2^;R26R* mice injected with tamoxifen (+Tam, A) or not (non-induced, B) to compare activation of the *Rosa26* reporter. A′ and B′ are magnified images of labeled cells from the same specimens. Arrowheads in B′ indicate labeled cells. (C) Distribution of normal cells subjected to spontaneous *mTmG* activation induced by leaky Cre recombinase. (D,E) Expected outcomes when tracing the progeny of BRAF-mutant cells, depending on downregulation of the *Cre* driver (D) and activation of *mTmG* before or after that of *Braf^CA^* (E). Shown are the sequence of recombination and the corresponding labeling patterns (1-3), of which ‘1.’ represents spontaneous reporter gene activation only. *Tg, thyroglobulin* transcript; *Cre*, *CreERT2* transcript. (F-H) Clonal expansion accompanying folliculogenesis in *TgCreER^T2^;Braf ^CA/+^;mTmG* mice at postnatal days 10 and 30 (P10 and P30, respectively). Thyroid images are from representative serial sections. Pre-follicular branching parenchyma (outlined) are shown in F, with inset showing DAPI-stained nuclei of an mGFP-positive (mG^+^) clone. Shown in G is a nascent oligoclonal follicle (encircled), with inset showing DAPI-stained nuclei corresponding to mG^+^ (closed dots) and mTomato-positive (mT^+^) follicular cells (closed dots), and surrounding cells (open dots) including a stromal cell (white open dot). Shown in H is a hyperplastic oligoclonal follicle (encircled). For comprehensive imaging of contiguous mG^+^ and mT^+^ epithelial domains (green and red arrow, respectively), see the stack series in Fig. S5. (I) Oligoclonal microcarcinoma involving clonal cooperativity of papillary growth. The small boxed area is shown magnified in panel I′, showing mT^+^ (top right), mG^+^ (bottom left) or DAPI (bottom right) fluorescence, or merged fluorescence (top left). Arrowheads indicate planar expansion of adjacent mG^+^ and mT^+^ clones. Asterisks indicate lumen of neoplasm; arrows indicate the transition zone of mT^+^ and mG^+^ epithelial domains. (J) Sketch, representing the large boxed area in I, indicating the lamellar pattern of oligoclonal growth. Scale bars: 500 µm (A,B), 100 µm (A′,B′,C,I) and 25 µm (F-H).

**Fig. 5.**
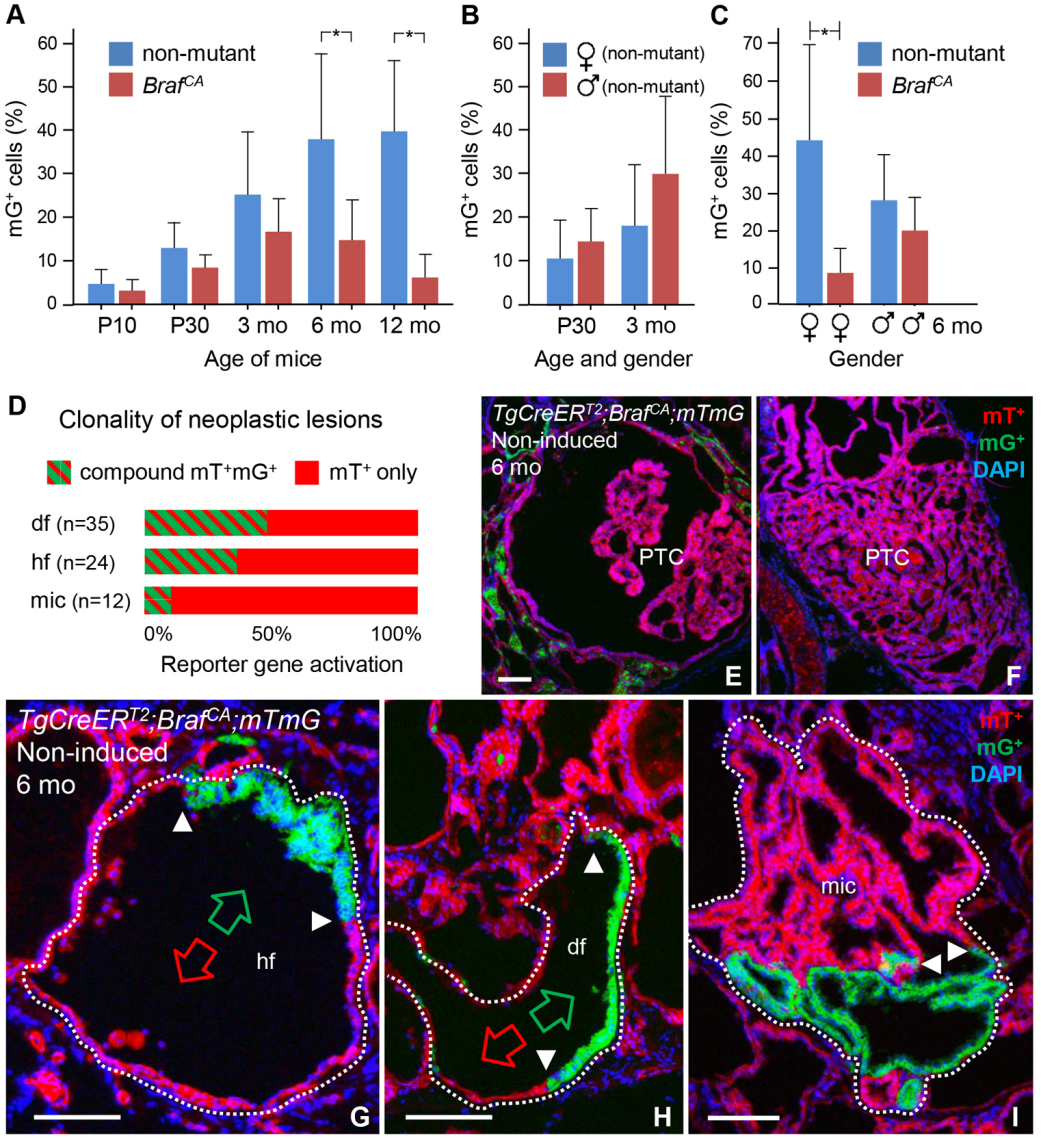
**Clonal selection of growth during thyroid tumorigenesis in*****TgCreER***^***T2***^***;Braf***^***CA/+***^***;mTmG*****mutant mice.** All data were obtained from animals not treated with tamoxifen. (A–C) Age- and gender-dependent changes in clonal expansion of mTomato-positive (mT^+^) and mGFP-positive (mG^+^) BRAF-mutant (*Braf*^*CA*^) cells. Clonal growth was estimated by counting mG^+^ cells in serial sections of the thyroid (three levels per lobe) – at postnatal days 10 and 30 (P10 and P30, respectively) and 3, 6 and 12 months (mo) of age – in mutant mice. The percentage of mG^+^ cells relate to the total number of DAPI-stained epithelial cells; thus, in every measurement, 100% comprises all encountered mG- and mT-labeled follicular and tumor cells. For each time point, the accumulation of mG^+^ thyroid cells is compared to that of age-matched *TgCreER*^*T2*^*;mTmG* (non-mutant) mice. The spontaneous rate of reporter gene activation (blue) and clonal expansion of mT^+^ versus mG^+^ mutant cells (red) are plotted in A; mean±s.d. (*P<0.005; *n*=6 for each group). Equal rates of reporter gene activation in non-mutant male (red) and female (blue) thyroids are plotted in B. Gender bias of mT^+^ versus mG^+^ clonal expansion in mutant mice (red) in comparison to the accumulation of mG^+^ cells in non-mutant mice (blue) is plotted in C; mean±s.d. (*P<0.005; *n*=3 for each group in B and C). (D) Predominance of lack of versus dual clonal reporter gene activation – revealed by uniform mT^+^ and compound mT^+^mG^+^ labeling, respectively – in neoplastic lesions in mutant mice aged 3 months. Horizontal bars indicate the mean relative values for each labeling and the type of lesion (*n*, indicated in graph) in total, based on five serially sectioned thyroids. Notice that lesions composed of only mG^+^ cells were not observed. (E,F) Papillary carcinomas with different tumor phenotypes – i.e. classic (E) and solid variant (F) – and without any signs of reporter gene activation in the tumor cells in 6-month-old mutant mice. (G-I) Representative images of dual-labeled lesions (encircled) with distinct (G,H) or indistinct (I) growth pattern of mT^+^ and mG^+^ clones. Arrowheads indicate clone borders; green and red arrows indicate contiguous mG^+^ and mT^+^ epithelial domains, respectively. hf, hyperplastic follicle; df, dilated follicle; mic, microcarcinoma; PTC, papillary thyroid carcinoma. Scale bars: 100 µm.

To get insight about the earliest stages of stochastic tumor development, *TgCreER^T2^;Braf^CA/+^* mice were crossed with established *Cre* reporter strains. Consistent with previous findings (Undeutsch et al., 2014), specificity of tracing the thyroid follicular lineage was confirmed by induced activation of Cre in *TgCreER^T2^* mice that carry a lox-STOP-lox lacZ allele targeted to the *Rosa26* (*R26R*) locus (Soriano, 1999). The follicular epithelium of *TgCreER^T2^;R26R* mice uniformly stained positive for X-gal corresponding to expression of Cre recombinase, whereas surrounding non-thyroid tissues were unlabeled (Fig. 4A,A′). In the absence of tamoxifen, only occasional X-gal-positive cells appeared in adult mice suggesting a low rate of spontaneous Cre-mediated recombination (Fig. 4B,B′). In similar experiments, the *mTmG* reporter designed to switch from membrane-tagged mTomato to mGFP expression upon activation was used to increase the resolution of lineage tracing (Muzumdar et al., 2007); hereafter, cells expressing either fluorescent label are referred to as mT^+^ and mG^+^ cells, respectively. This revealed a gradual accumulation of mG^+^ cells from birth eventually comprising almost 40% of the follicular cell population in adult *TgCreER^T2^;mTmG* mice (Figs 4C and 5A). The fact that, in aging mice, spontaneous reporter activation leveled off might relate to the accumulation of inactive follicles, comprising cells with diminished function that probably exhibit reduced *Tg* promoter activity (Studer et al., 1978). Nonetheless, since *mTmG* faithfully detected contiguous mG^+^ progenies of normally dividing follicular cells (Fig. 4C), we chose this reporter strain for clonal analysis of BRAF-induced tumor development.

Unlike after induction, it is conceivable that, on the basis of non-parallel recombination, spontaneous Cre-mediated activation of multiple alleles in a given cell occurs independently and sequentially, most probably with a considerable lag time (Liu et al., 2013). We found that *Braf^CA^* activation concomitantly downregulates TG and Cre (Figs 3C and 4D). Therefore, the prediction is that, in *TgCreER^T2^;Braf^CA/+^;mTmG* mice – if the reporter gene or *Braf^CA^* is being first activated – mutant cells become either mGFP-labeled or retain mTomato expression resulting in clonal expansion of mG^+^ or mT^+^ cell cohorts, respectively (Fig. 4E). To confirm this assumption and validate the usefulness of the model in clonal tracing, serially sectioned thyroids obtained from non-induced *TgCreER^T2^;Braf^CA/+^;mTmG* mice where carefully examined from the onset of *Tg* expression in embryonic development – i.e. ∼E15.5 – and spanning the entire period of postnatal growth until the majority of thyroid cells become senescent in adulthood, prevailing also in old mice.

The embryonic thyroid normally consists of a branching network of parenchyma in which folliculogenesis occurs from embryonic day E15.5 onwards (Liang et al., 2018). This did not change in mutant mouse embryos (investigated at E18.5). We also found that mG-labeled cells were rare at this early stage (data not shown). However, postnatally, the number of mG^+^ cells increased, the majority showing a random distribution consistent with stochastic reporter activation (Fig. S5A). Additionally, mG^+^ cells clustered both in the pre-follicular parenchymal cords and in nascent follicles, suggesting initiation of clonal expansion (Fig. 4F,G). There were no signs of abnormal follicular growth at postnatal day 10 (P10).

At the age of 1 month, the most conspicuous finding in *TgCreER^T2^;Braf^CA/+^;mTmG* mice was the presence of compound follicles consisting of both mT^+^ and mG^+^ cells (hereafter referred to as mT^+^mG^+^ follicles/lesions) that were considerably larger than the surrounding normal-sized follicles (Fig. 4H and Fig. S5B^1^-B^6^). The dual-labeled follicles consisted mostly of two distinctive mT^+^ and mG^+^ epithelial domains of equal size, which were contiguous and encircled an irregular lumen (Fig. 4H and Fig. S5B^1^-B^6^). Some enlarged follicles uniformly consisted of mT^+^ cells, whereas follicles predominated by mG^+^ cells were only rarely observed. In 3- to 6-month-old mice, compound follicles coexisted with similarly dual-labeled microcarcinomas, consistent with the coordinated expansion of mT^+^ and mG^+^ tumor clones (Fig. 4I). Notably, mT^+^ and mG^+^ portions of the tumorous epithelium were always interlinked at discrete sites, indicating a shared follicle origin (Fig. 4I; Fig. S6A). Both clones, thus, participated in the generation of a conspicuous papillary growth pattern by means of multiple folding of the single-layered epithelium (Fig. 4I,I′,J; Fig. S6A-C). This indicated that tumor cell proliferation virtually always occurred by planar cell division, implying that the originating epithelium maintained its apicobasal polarity and did not stratify owing to a multilayering process. Tumor-associated stromal cells were sparsely present, intervening adjacent layers of the neoplastic epithelium (Fig. 4I′; Fig. S6B′).

These experiments, thus, enabled us to trace novel features of tumor evolution in mouse PTC confined to a single follicle origin. Neoplastic follicles developed into tumors by coordinated growth of BRAF-mutant cells in an oligoclonal fashion.

### Tumor evolution involves clonal selection of cells with early onset Braf^CA^ activation

A predominance of mT^+^ cells over mG^+^ cells was evident in most tumorous lesions (Fig. 4I; Fig. S6). To estimate the overall clonal contribution, we morphometrically quantified the relative number of mG^+^ cells accumulating in the thyroid of *TgCreER^T2^;Braf^CA/+^;mTmG* mice aged between P10 and 12 months (*n*=27; each lobe serially-sectioned at three levels). To exclude that calculations were biased towards stromal cells, the number of which varied among lesions, only parenchymal cells, distinguished by their epithelial shape, were counted. This showed that the proportion of mG^+^ cells in mutant mice never exceeded the corresponding number in age-matched *TgCreER^T2^;mTmG* control mice (Fig. 5A). By contrast, numbers of mT^+^ cells in mutants gradually increased at the expense of mG^+^ cells, the latter of which comprised <10% in 12-month-old mutants (Fig. 5A). Moreover, although at age P30 and 3 months the spontaneous rate of Cre-mediated recombination was similar in both sexes (Fig. 5B), expansile mT^+^ clones predominated in female mice (Fig. 5C), consistent with the observed sex differences in tumor growth (Fig. 1C). Dual-labeling analysis of individual lesions further indicated that clonal selection in favor of mT^+^ mutant cells already started during early tumor development (Fig. 5D). Accordingly, the majority of manifest PTC tumors – independently of subtype – consisted exclusively of mT^+^ cells (Fig. 5E,F). Neoplasms solely composed of mG^+^ cells were not encountered at any age.

It is noteworthy that mT^+^ and mG^+^ clones coexisting in a single enlarged follicle occasionally showed distinct growth characteristics (Fig. 5G,H). However, some dual-labeled tumors displayed a homogeneous growth pattern for which the participating clones were, in fact, impossible to distinguish without clonal tracing (Fig. 5I and Fig. S6A-C). These observations further argue that the cellular response to mutant BRAF differs much among follicles.

Next, we quantified the clonal responsiveness to vemurafenib administered to *TgCreER^T2^;Braf^CA/+^;mTmG* mice from weaning up to 3 months of age (Fig. S7A, top). This showed that treatment with PLX4720 largely restored *mTmG* activation (Fig. S7A, bottom). This effect is likely to be largely dependent on recovery of Cre expression in BRAF-mutant cells. Accordingly, mG^+^ cells accumulated in follicles that retained normal size and shape (Fig. S7B,C), suggesting that most mutant cells maintained a quiescent state. Nonetheless, drug-treated mice occasionally developed thyroid microcarcinomas that were essentially free of mG^+^ cells (Fig. S7C). This indicated that a subpopulation of mT^+^ mutant cells gained a growth advantage – presumably before drug administration – and that the expanding clones had already acquired resistance to vemurafenib at a pre-tumorous stage.

### Restricted growth response of BRAF-mutant cells coexisting with non-mutant follicular cells

**Fig. 6.**
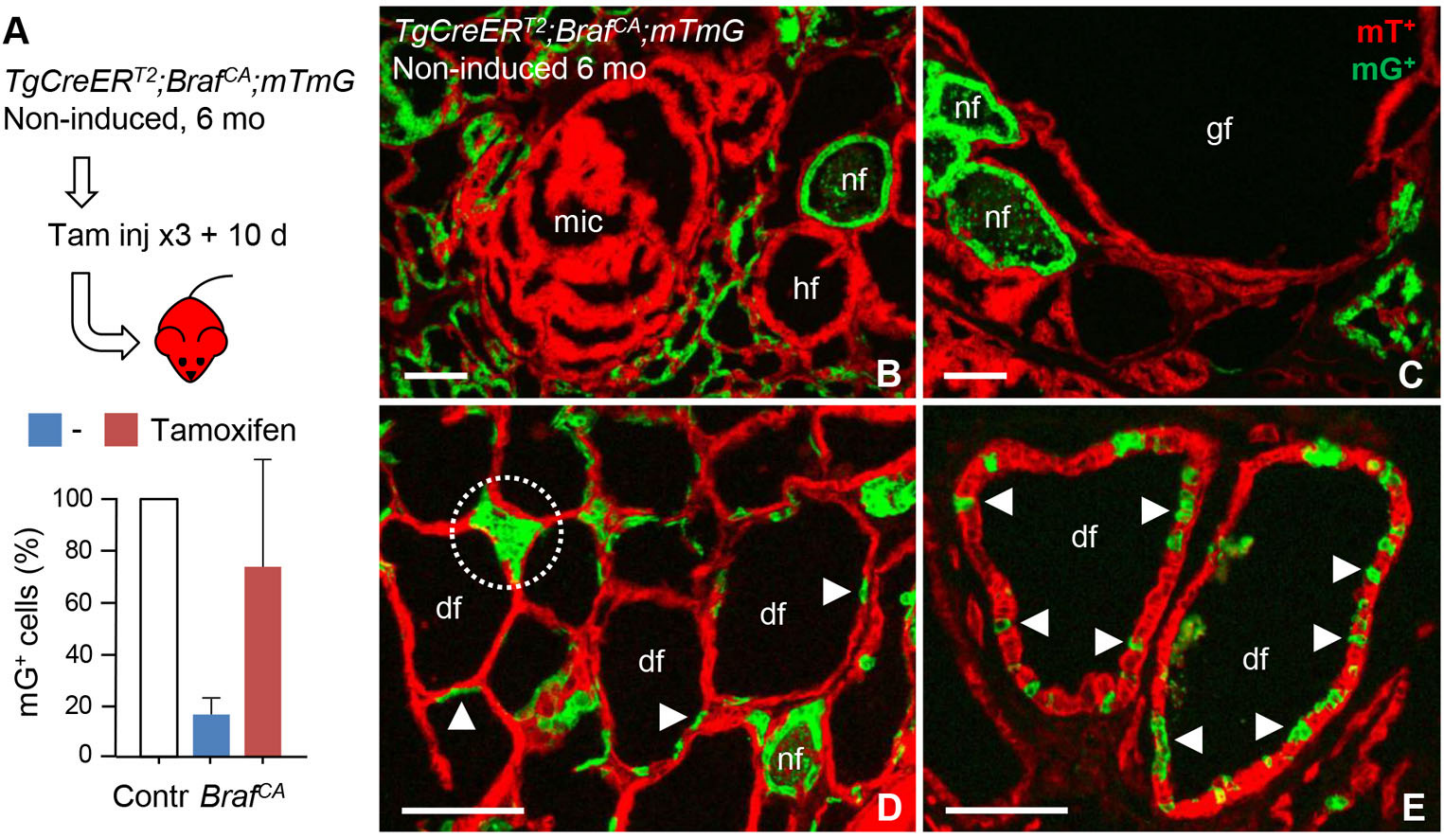
**Follicular distribution of BRAF-mutant thyroid cells outside tumors.** All data refer to induced reporter gene activation in 6-month-old *TgCreERT2;BrafCA/+;mTmG* mice – to distinguish between unlabeled mutant cells and responding non-mutant cells that comprise preserved Cre activity – which already developed neoplastic lesions due to spontaneous Cre-mediated recombination. (A) Schematic, depicting experimental set-up, and morphometric quantification of responding cells that recombined *mTmG*. (Top) Mutant mice were injected intraperitoneally with tamoxifen (10 mg/ml; 50 µl once every day for three consecutive days followed by analysis 10 days (10 d) later. (Bottom) Numbers of mGFP-positive (mG^+^) cells in mutant mice treated with tamoxifen (red) or not (blue) compared with the corresponding spontaneous activation levels in age-matched control (Contr) *TgCreER*^*T2*^*;mTmG* mice (set to 100%). Analysis was for three section levels per gland; mean±s.d. Contr (*n*=6), untreated mutants (*n*=3), tamoxifen-treated mutants (*n*=7). (B-E) Density of mG-labeled cells within follicles of varying size and shape. Encircled area in D indicates a compacted follicle consisting of only induced, non-mutant cells; its abnormal shape likely depends on crowding from the neighboring dilated follicles. Arrowheads indicate single or clustered mG+ cells. nf, normal follicle; hf, hyperplastic follicle; df, dilated follicle; gf, giant follicle; mic, microcarcinoma. Scale bars: 100 µm.

The abundance of moderately enlarged follicles that fully responded to vemurafenib and yet did not develop tumors suggested that BRAF-mutant cells ceased to proliferate soon after oncogenic activation. To distinguish mutant from non-mutant cells in these follicles, 6-month-old *TgCreER^T2^;Braf^CA/+^;mTmG* mice were daily injected (3×) with tamoxifen 10 days before sacrifice (Fig. 6A, top). Delayed induction of Cre increased the number of mG^+^ cells to ≈75% of that generated by spontaneous reporter activation in control mice (Fig. 6A, bottom). This provided a reliable estimate of the relative proportions of responding non-mutant cells and non- responding mutant cells at 6 months. It should be noticed that, in tissue sections, induced mG^+^ cells had a markedly heterogeneous distribution. Normal-sized follicles were either uniformly or partially labeled, whereas hyperplastic follicles, giant follicles and microcarcinomas were virtually devoid of mG^+^ cells (Fig. 6B,C). This confirmed that tamoxifen was unable to trigger reporter gene activation in neoplastic lesions but also that seemingly normal follicles retained BRAF-mutant cells in the encircling epithelium. Of particular interest is that many of the moderately enlarged follicles contained only occasional mG^+^ cells (Fig. 6D) or consisted of alternating mG^+^ and mT^+^ epithelial segments (Fig. 6E).

In this experiment, tamoxifen is likely to have induced co-activation of *Braf^CA^* and the reporter in normal cells with maintained Cre expression. However, the lag time between injection and sacrifice was too short to generate significant clonal expansion of newly induced cells. Moreover, all induced cells were probably encountered in the mG^+^ population. Altogether, this indicated that a large number of BRAF-mutant cells that coexist with non-mutant cells were growth-restricted and unable to elicit the entire carcinogenic cascade.

## DISCUSSION

By means of spontaneous Cre-mediated activation of oncogenic *Braf^CA^* in the absence of tamoxifen, we were able to monitor sporadic PTC development from the earliest signs of tumor initiation in infancy to the occurrence of macroscopic tumors in adult mice. The random activation of *Braf^CA^* in a limited number of cells accumulating over time distinguished different neoplastic growth patterns depending on spatiotemporal cues inherent to the morphogenesis and tissue organization of the thyroid. By providing a secluded microenvironment for neoplastic growth, each follicle constitutes a minimal effective tumorigenic unit. However, within a single follicle, the relationship between simultaneously occurring BRAF-mutant and non-mutant cells seems to determine whether cooperation or competition mechanisms facilitate or counteract tumorigenesis in an autonomous fashion. The follicular architecture, thus, identifies and delineates intrinsic boundaries of the cancerization field in the thyroid. Our study also provides the first experimental insight into morphogenesis of the archetypal papillary growth pattern of PTC. Manufactured by oriented cell division – i.e. longitudinally with reference to the curved plane of the originating follicle – proliferating clones expand linearly and give rise to incremental folding of the epithelium. This growth mechanism, by which tumor cells maintain both apical polarity and a basal border towards the extrafollicular space, clearly distinguishes PTC from FTC that is characterized by the separation of groups of cells required to generate a multitude of neoplastic follicles. How such divergent tumor growth patterns arise from constitutive activation of the same signaling pathway, driven by *BRAF* and *RAS* mutations in human thyroid cancer cells, remains a key question.

Non-parallel recombination of *Braf^CA^* and the *mTmG* reporter due to leaky Cre activity was instrumental in distinguishing BRAF-mutant thyroid cells of different clonal origin. A supporting concept for our conclusions is also that, in this model, inducible expression of Cre is permanently shut-off once *Braf^CA^* is activated, preventing further recombination events in the cell progeny. In support of this assumption, TG and Cre were coordinately downregulated in mutant cells and recovered in response to treatment with PLX4720. This confirms previous reports indicating that *Tg* promoter activity in mouse thyroid follicular cells is fully repressed as long as mutant BRAF kinase activity prevails (Chakravarty et al., 2011; Charles et al., 2011). This strong and persistent dedifferentiating effect with reference to thyroid function probably relates to the fact that, unlike constitutively active RAS signaling, activation of the MAPK signaling pathway through BRAF^V600E^ resists feedback inhibition (Pratilas et al., 2009). It is, therefore, conceivable that any TG and Cre immunoreactivity occasionally found in neoplastic lesions is derived from normal cells entrapped in the tumor rather than from mutant cells. Although we neither can technically exclude that some tumor cells retained TG expression nor the possibility of them subclonally labeled due to leaky Cre, it is highly unlikely that *mTmG* activation coincided with the initiation of a putative subclone with different growth features. Moreover, if reporter activation were to occur subclonally it is likely to generate more-random mG^+^ labeling than evident in most neoplastic lesions. In fact, induced reporter activation in response to delayed tamoxifen administration predominantly – if not exclusively – labeled follicular cells that exhibited no signs of clonal growth. Thus, in our working model, it is conceivable that progenies of mG^+^ ancestral mutant cells are inevitably labeled, simply because reporter gene activation occurred before activation of *Braf^CA^*, and that mT^+^ tumor clones are unable to undergo further Cre-mediated recombination events after *Braf^CA^* is activated (Fig. 4E).

The most-conspicuous finding of our dual reporter tracing experiments was the oligoclonal nature of individual tumors. This was unexpected in view of current understanding that PTC in humans is a monoclonal disease. The concept of monoclonality of PTC relies mainly on genomic analysis from the TCGA study (Cancer Genome Atlas Research Network, 2014) and paired sequencing of primary tumors and distant metastases (Giannini et al., 2007; Gopal et al., 2018; Ricarte-Filho et al., 2009). This indicates that the genetic drivers of thyroid tumorigenesis are mutually exclusive and that their identity is maintained during cancer progression in the majority of cases. However, as recently reviewed by Fugazzola et al., the bulk of information on intratumoral genetic heterogeneity characterizing earlier stages of PTC call for an unbiased evaluation of this controversial issue (Fugazzola et al., 2020). More specifically, findings of a heterogeneously distributed *BRAF* mutation that is confined to a subset of tumor cells in small-size tumors (de Biase et al., 2014; Finkel et al., 2016) suggest that monoclonality for *BRAF* predominating in more-advanced stages of PTC is the result of tumor evolution. Inconsistent immunostaining of BRAF^V600E^ in a minority of manifest PTCs (Ghossein et al., 2013) is in line with this possibility. Our findings that mT^+^ tumor clones have a growth advantage over mG^+^ clones, outrivaling them over time, provide the first experimental proof regarding clonal selection of mutant cells in a mouse model of differentiated thyroid cancer. In humans, a homogeneous pattern of X chromosome inactivation (XCI) among tumor cells, which characterizes and distinguishes individual tumor loci of multifocal PTCs (Shattuck et al., 2005), is often referred to when refuting an oligoclonal origin of thyroid cancer. However, patch size confined to the progeny of a single precursor cell inheriting the same XCI pattern is so large in human thyroid tissue that, on this basis alone, assessment of clonality is doubtful (Jovanovic et al., 2003; Novelli et al., 2003). Homotypic expression of X-linked markers may, in fact, conceal a multiclonal tumor origin from detection (Garcia et al., 2000; Parsons, 2018).

As documented in studies regarding aggregation chimeras of *Apc* mutant mice, a multi- or polyclonal tumor origin is evident in hereditary intestinal cancer (Halberg and Dove, 2007). In this model, recruitment through facilitating transformation of neighboring crypt cells rather than cooperation of two independent progenitors appears necessary for tumors to thrive (Thliveris et al., 2013). A similar recruitment mechanism characterizes a mouse skin cancer model in which pre-malignant lesions develop polyclonally but the arising carcinoma evolves through a clonal sweep that favors the *Hras* mutant tumor-initiating cells (Reeves et al., 2018). In our present study, we cannot exclude the possibility that non-mutant thyroid follicular cells similarly contribute to phenotypic changes elicited by activation of *Braf^CA^*. However, the experiments with delayed induction in response to tamoxifen showed that normal responding cells coexist with mutant cells in the same follicle without signs of clonal expansion – which argues against recruitment as a predominant factor. On the contrary, most neoplastic lesions exclusively contained cells that failed to recombine *mTmG*, which is consistent with *Braf^CA^* activation that had already occurred in all those tumor cells prior to tamoxifen treatment. Rather, the heterogeneous growth pattern of oligoclonal thyroid tumors, indicates that the participating mutant clones, distinguished by lineage tracing, are likely to have a dual ancestral cell origin.

In view of the fairly high rate of spontaneous Cre-mediated recombination in *TgCreER^T2^* mice, eventually comprising the majority of follicles and several cells in each follicle, the number of overt tumors encountered in adult *TgCreER^T2^;Braf^CA^* mice was surprisingly low. Assuming that Cre recombines *Braf^CA^* and *mTmG* alleles with similar efficiencies, and taking into account recent flow cytometry data obtained from studies using normal mice (Gawade et al., 2016), it is estimated that 16,000 thyroid cells express BRAF^V600E^ in mutant adult mice. Even if this were an overestimate, it would still be conceivable that only a small percentage of primarily BRAF-mutant cells are fully transformed and capable to generate tumors in the current model. From this follows that most follicular cells resist oncogenic activation by mutant BRAF, suggesting the intriguing possibility that mouse thyroid tissue possesses a natural ability to restrain the behavior of precancerous cells. Such tumor-suppressive signals may serve to maintain epithelial differentiation and tissue homeostasis (Bissell and Hines, 2011), e.g. by discarding potentially harmful cells through apical extrusion (Kon et al., 2017; Vermeulen et al., 2013). Intrinsic surveillance mechanisms might thus explain why tumors did not develop in a fraction of thyroid cells when *Braf^CA^* activation was mediated by administering viral vectors directly into the thyroid gland, as previously reported (Shimamura et al., 2013). Nonetheless, by using another *Cre* driver able to mediate a higher transformation efficiency, intrathyroidal vector injections generated PTC with phenotypic characteristics similar to those described here, although with a much delayed onset (Shimamura et al., 2018). This suggests that increased BRAF^V600E^ expression is sufficient to overcome the natural protection against oncogenic insults that prevail in the mouse thyroid. Further characterization of heterogenous tumor development in our current model might be accomplished by adopting BaseScope^TM^ *in situ* detection of driver point mutations, like *BRAF V600E*, as recently developed for human tumor tissues (Baker et al., 2017).

Consistent with Mina Bissell's dictum “the phenotype is dominant over the genotype of even tumor cells”, which explains the dormancy of occult tumors (Bissell and Hines, 2011), normal thyroid follicular cells thus appear to exert a stabilizing influence on the quiescent status of a neighboring cell that carries a potentially oncogenic mutation. This raises the obvious question which cellular mechanism(s) overcome the protective role of the microenvironment provided by mature follicles to elicit tumorigenesis in the thyroid. TSH have been shown to release BRAF-mutant cells from oncogene-induced senescence (Zou et al., 2016), presumably related to the fact that supraphysiological levels of TSH are mitogenic and stimulate thyroid cell proliferation. A related clue, supported by our present work, may be sought for in the dynamic period of thyroid development. Previous reports indicate that immature thyroid cells are more susceptible to transformation, presumably owing to their high mitotic rate during infancy, than adult thyroid cells, which are characterized by replicative senescence (Coclet et al., 1989). This, in fact, correlates with the risk of developing thyroid cancer after radiation exposure (Saad et al., 2006). As evidenced by the surge of childhood PTCs reported after the Chernobyl fallout in 1986 (Williams, 2008), this relationship probably explains the remarkably high radiosensitivity of the thyroid gland in children. Based on data obtained from ultrasound screening implemented after the Fukushima nuclear accident (Yamashita and Suzuki, 2013), there is now compelling evidence to suggest that tumor initiation of also sporadic PTC occurs much earlier than previously recognized (Williams, 2015). Notably, *BRAF* mutations predominate in PTCs of the younger members of the Japanese population, which differ genetically from radiation-induced thyroid cancer (Mitsutake et al., 2015). On this basis, a revised hypothesis regarding thyroid carcinogenesis in humans has been proposed (Williams, 2015), arguing that tumor initiation already starts in infancy and that microcarcinomas may be dormant for decades or remain undiagnosed, as evident from autopsy materials (Harach et al., 1985).

We believe our study here provides direct experimental support of Williams’ hypothesis. The earliest signs of dual-labeled pre-neoplastic lesions coincided with perinatal folliculogenesis. Note that normal follicles are polyclonal by nature (Ma et al., 2010; Novelli et al., 2003; Thomas et al., 1989), as they arise from multiple precursor cells that assemble during the final stage of thyroid morphogenesis (Nilsson and Fagman, 2017). The foundation to develop oligoclonal tumors, thus, exists in newly formed follicles. It is important to also consider that, *in vivo*, naïve thyroid progenitors start to differentiate before a follicle lumen is discernible (Liang et al., 2018). For TG, to be retained or not in the lumen of neoplastic lesions, as evident in this study, thus provides a plausible means to estimate the fate of BRAF-mutant cells with temporally different onset of oncogenic activation. Lesions entirely devoid of TG may have been initiated already at a pre-follicular stage of cells, whereas TG-containing lesions – which rarely progressed to cancer – were most likely to have emerged from mature follicles. A related issue concerns the recruitment of cells when forming a follicle. As assembly of a basement membrane is required for folliculogenesis (Villacorte et al., 2016), it can be assumed that, once an enclosing basement membrane is established, further cells from the outside cannot join and that all cells populating a single follicle are likely to descend from few, perhaps only two, initiating progenitor cells (Nilsson and Fagman, 2017). From this, it follows that the earlier a driver mutation is activated the greater the chance of obtaining a growth advantage over non-mutant cells present in the same follicle. Therefore, oncogenic co-activation of immature cells and clonal cooperation of growth, put into effect at a crucial phase of thyroid development, might facilitate tumor initiation by escaping the redundancy of normal cells in mature follicles. In our present model, the documented growth advantage of mT^+^ tumor clones – which only require one recombination event – might similarly be related to early onset of oncogenic activation.

The prevalence of *BRAF* mutations that potentially contribute to carcinogenesis in normal thyroid tissue is unknown. However, the fact that PTC is frequently multifocal, with independent clonal origin of individual lesions (Giannini et al., 2007; Lu et al., 2016; Shattuck et al., 2005), and that ≥50% of all PTC cases comprise a mutated *BRAF* gene (Howell et al., 2013; Xing, 2005), argues that the sporadic mutational rate is significant. It is noteworthy that multicentric PTCs diagnosed in a single individual are often classified as distinct histopathological variants (Howell et al., 2013; Xing, 2005). In our current model, most PTC subtypes were evident in mutant mice aged 12 months or older, suggesting that subclonal events are involved. However, at least the classic and cystic PTC variants could be traced back to neoplastic follicles with different properties and distribution in young animals. This suggests that the natural follicle heterogeneity influences the pattern of BRAF-driven tumor growth in a way that is similar to that of multinodular goiter development (Kopp et al., 1994; Peter et al., 1985). To get further insight, it is important to consider that thyroid cells do not uniformly respond to goitrogenic stimulation, which constrains growth to susceptible follicles or even segments of the follicular epithelium that are more sensitive to growth stimuli – and perhaps prone to cancerous growth – than most other thyroid cells (Smeds et al., 1987). The foundation of a differential growth response is probably generated embryonically, as thyroid progenitor cells with a high intrinsic growth rate separate from cells that undergo differentiation (Liang et al., 2018). It is assumed that segregation of cells with different growth properties during development generates follicles of different size and tissue distribution in the adult gland (Nilsson and Fagman, 2013). Developmental trajectories that govern follicle heterogeneity might, therefore, add to the factors that modify cellular responses to mutant BRAF in the mouse thyroid. Validity of this hypothesis gains support from recent multicolor lineage tracing of xenografted colorectal cancer cells, which indicate that clone location and tissue geometry may contribute to heterogeneous clonal expansion (van der Heijden et al., 2019).

A female bias in the formation of thyroid tumors is evident in mice comprising a conditional inactivation of *Pten* (Antico-Arciuch et al., 2010; Yeager et al., 2007). This model is characterized by goiter and the susceptibility to develop thyroid cancer due to increased activation of the PI3K/AKT pathway, which sidelines the natural obstruction of thyroid growth through an estrogen-dependent mechanism. It is of particular interest that the co-inactivation of *Pten* and *Cdkn1b*, the latter of which encodes cyclin-dependent kinase inhibitor 1B (CDKN1B), further accelerates thyroid growth that is similar in both sexes (Antico-Arciuch et al., 2010). This suggests that stimulation of thyroid cell proliferation above a certain level abolishes possible gender-based differences, which might explain why mice comprising constitutively active *Braf^CA^* show no sex differences regarding penetrance, latency or severity of PTC (Franco et al., 2011). Our study complies with this concept, indicating that BRAF^V600E^ expression in a minority of cells generates larger thyroid tumors in females than in males. Our findings further suggest that tissue microenvironment, possibly involving local interplay of BRAF-mutant and non-mutant cells, modifies thyroid tumor development through the action of estrogen or other sex-related factors.

**Fig. 7.**
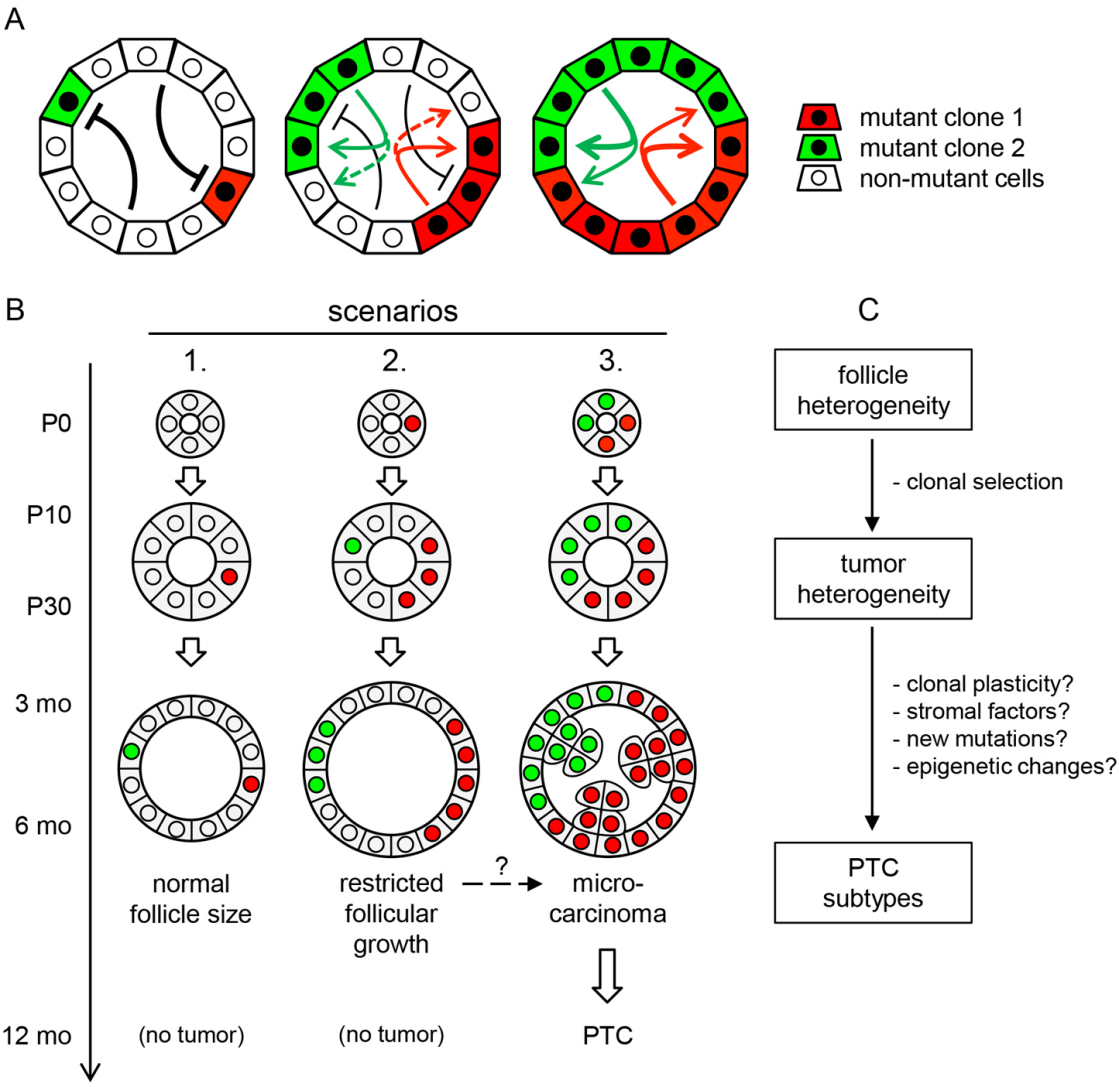
**Clonal evolution of tumor development and heterogeneity in sporadic thyroid cancer, as proposed from the presented *TgCreER^T2^;Braf^CA/+^* mouse model.** (A) Constraints on tumor initiation depend on cell composition of thyroid follicles. Red and green arrows indicate suggested clonal cooperativity of mutant clones (solid lines) and putative bystander inhibitory effects of oncogenic activation on non-mutant cells (dashed lines). Supernumerary non-mutant cells are likely to exert an inhibitory action on single mutant cells, preventing oncogenic transformation and tumor initiation (inhibitory arrows). (B) Three scenarios of restricted or promoted neoplastic growth depending on spatiotemporal onset of oncogenic activation related to follicle maturity. The three scenarios are based on timing and coincidence of two independent oncogenic mutant clones (depicted in red and green). (C) Spatial factors related to natural follicle heterogeneity can influence BRAF^V600E^-induced tumor development and, ultimately, the PTC tumor phenotype. Clonal selection of growth is an early event in tumorigenesis. Tumor progression to overt cancer is likely to involve additional clonal and subclonal alterations. See Discussion for further comments.

In summary, we provide a new mouse model to understand the earliest stages of sporadic cancer development in the thyroid gland, and uncover *in vivo* mechanisms related to tissue maturation and architecture that restrain or markedly modify the oncogenic response. In our model, spontaneous Cre-mediated recombination formed the basis of stochastic *Braf^CA^* activation, which mediates random expression of BRAF^V600E^ in few cells, one at a time. As a result, the prevalence of BRAF-mutant thyroid cells slowly increased from the onset of functional differentiation and folliculogenesis in embryonic life to eventually accommodate the majority of mature follicles in adults. Concurrent observations of constrained and promoted tumorigenesis that arise independently albeit in close proximity within mouse thyroid tissue are consistent with a heterogeneous response to mutant BRAF, depending on spatiotemporal factors (Fig. 7). The key to understanding this is the autonomous nature of single, individual follicles, which consist of a defined but variable number of epithelial cells related to follicle size; i.e. what takes place in one follicle does not directly influence the neighboring ones. This means that the fate of a follicular cell carrying a potentially oncogenic mutation relies on its ability to overcome surveilling mechanisms mediated by non-mutant cells that are confined to the originating follicle (Fig. 7A). It, therefore, follows that coincidental activation of multiple mutant cells when follicles are about to form would stand a much better chance of initiating tumor development than a single mutant cell that resides in the follicular epithelium predominated by normal cells (Fig. 7B). We also found evidence of a clonal foundation of inter- and intratumor heterogeneities, which sheds light on the puzzling issue of how PTC subtypes designated by distinct histopathological features might develop from lesions that harbour identical driver mutations. Divergent growth patterns can be traced back to the follicle origin, suggesting that properties inherent to the generation of natural follicle heterogeneity influence tumor development (Fig. 7C). Clonal selection and subclonal genetic alterations may further modify the generation of tumor phenotype diversity in differentiated thyroid cancer.

## MATERIALS AND METHODS

### Mouse lines and treatments

*TgCreER^T2^* mice with inducible Cre under control of the *Tg* promoter (Undeutsch et al., 2014) were crossed with *Braf^CA^* mice (Dankort et al., 2007) to generate *TgCreER^T2^;Braf^CA/+^* mice. Both *Cre* lines were crossed with *Rosa26R* and *mTmG* reporter mice for lineage tracing, as described (Muzumdar et al., 2007; Soriano, 1999). Strains were backcrossed with C57BL/6J mice at least ten generations before recombination. Tail tips were sampled for genotyping with PCR. Tamoxifen dissolved in sunflower oil (10 mg/ml) was injected intraperitoneally (50 µl) once daily for a total of 3 days to induce expression of CreER^T2^. PLX4720 (417 ppm), the precursor compound of vemurafenib (PLX4032), and control dietary pellets (both provided by Plexxikon) were continuously supplied during the treatment period. Animal experiments were approved by the regional ethic committee (Dnr 26-2013 and Dnr 5.8.18-03925/2018) according to European standards and national regulations provided by the Swedish Agriculture Agency.

### Histology, X-gal staining, immunohistochemistry and fluorescence microscopy

After euthanasia, thyroids excised en bloc with the trachea, esophagus and muscles were fixed in 4% paraformaldehyde, and processed for paraffin sections and routine hematoxylin & eosin (H&E) staining. For X-gal staining, fixed samples were washed in 2 mM MgCl_2_, 0.1% sodium deoxycholate, 0.02% Nonidet P-40 in PBS pH 7.3 (washing buffer), incubated overnight at 37°C in 7.2 mM NaCl, 5 mM K_3_Fe(CN)_6_, 5 mM K_4_Fe(CN)_6_ and X-gal (Sigma-Aldrich) dissolved in washing buffer (X-gal-staining solution), washed again and fixed before embedding into paraffin. For immunohistochemistry (IHC), deparaffinized sections were subjected to epitope retrieval by PT Link (DacoCytomation) and quenching of endogenous peroxidase activity prior to immunostaining was optimized using the Dako EnVision system. Antibodies used were rabbit polyclonal anti-human thyroglobulin/TG (cat. no.: A0251, 1:5000; DakoCytomation), rabbit monoclonal anti-Cre recombinase (cat. no.: 15036, 1:125; Cell Signaling), rabbit monoclonal anti-mouse CK19 (cat. no.: ab133496, 1:1800; Abcam) and rabbit polyclonal anti-mouse NKX2-1 (cat. no.: PA0100, 1:1000; BioPat); and the Rat Impress system for rat monoclonal anti-mouse E-cadherin/CDH1 (cat. no.: 13-1900, 1:4000; Novex, Life Technologies). Immunostaining of human BRAF^V600E^ was performed under both acidic and basic conditions according the manufacturers’ instructions, using mouse monoclonal against human VE1 (cat. no.: E19290, between 1:100 and 1:500; Spring Bioscience) and rabbit monoclonal anti-human RM8 (cat. no.: SAB5600047, between 1:100 and 1:500; Sigma-Aldrich). Sections were viewed and imaged using an Olympus BX45TF microscope equipped with a Nikon DS-U2 camera. For evaluation of *mTmG* activation, fixed tissue samples were incubated overnight in 30% sucrose, embedded in OCT Tissue-Tek (Sakura, Zoeterwoude, The Netherlands) and saved at −80°C. Cryosections were collected on Super Frost glass slides (Vector, Burlingame, MA) and counterstained with DAPI nuclear stain (Sigma-Aldrich) before mounting with fluorescence mounting medium (DakoCytomation). Fluorescence was analyzed using a Zeiss Axioskop2 plus microscope equipped with a Nikon DS-Qi1Mc camera. Image acquisition and processing was carried out using the NIS Elements Imaging Software.

### Quantitative real-time PCR

Mouse thyroid samples stored at −80°C in RNA*later*™ solution (Thermo Fisher Scientific) were homogenized using a TissueLyser II (Qiagen) and RNA-extracted using the RNeasy mini kit (Qiagen) according to manufacturer's instructions. Final RNA amounts were determined spectrophotometrically (NanoDrop1000; Thermo Scientific) after cDNA had been synthesized by using TATAA GrandScript cDNA Synthesis Kit (TATAA Biocenter) and T100 Thermal Cycler (Bio-Rad) and stored at −20°C. Primers to mouse *Tg* (forward: 5′-CATGGAATCTAATGCCAAGAACTG-3′; reverse: 5′-TCCCTGTGAGCTTTTGGAATG-3′), *Slc5a5* (forward: 5′-TCCACAGGAATCATCTGCACC-3′; reverse: 5′-CCACGGCCTTCATACCACC-3′), *Tpo* (forward: 5′-CAAAGGCTGGAACCCTAATTTCT-3′; reverse: 5′-AACTTGAATGAGGTGCCTTGTCA-3′), *Tshr* (forward: 5′-TCCCTGAAAACGCATTCCA-3′; reverse: 5′-GCATCCAGCTTTGTTCCATTG-3′), *Pax8* (forward: 5′-GATAGGAGACTACAAGCGGCA-3′; reverse: 5′-CGGATGATTCTGTTGATGGAGC-3′), *Erα1* (forward: 5′-AGGGAAATCTTGAGCCCCTA-3′; reverse: 5′-ACACACCCTACAGCCCTCAT-3′) and *ER^T2^* (forward: 5′-ATGATTGGTCTCGTCTGGCG-3′; reverse: 5′-CCAGGAGCAAGTTAGGAGCAA-3′) were designed applying Primer-BLAST (Ye et al., 2012) based on sequences retrieved from public databases (Ensembl or Santa Cruz Genome Browser). BLAST analysis verified that the selected primer sequences were sufficiently different from the rest of the mouse transcriptome. *In silico* oligonucleotide secondary structure prediction was performed with NetPrimer (PREMIER Biosoft International). Specificity of primers was confirmed by agarose gel electrophoresis of amplicons. A reference gene panel for mouse (TATAA Biocenter) was evaluated with NormFinder; *Gapdh* was chosen as optimal reference gene on the basis of predefined software criteria for relative quantification. Quantitative real-time PCR (qPCR) was performed using a CFX384 Touch real-time cycler (Bio-Rad) and TATAA SYBR GrandMaster Mix (TATAA Biocenter), 400 nM primer and 2 μl cDNA. Formation of expected PCR products was confirmed by agarose gel electrophoresis and all samples were analyzed by melting-curve analysis.

### Morphometry

The thyroid gland *in situ* (*n*=82) was photographed together with a ruler using an iPhone6 in-built camera. Lobe longitudinal and transverse diameters were measured with a digital slide gauge. Lobe volume was calculated using the standard formula for ellipsoids, *e*=*Height *× *Width *× *Depth*×(π/6), as reported (van Isselt et al., 2003). Transverse diameter was used to estimate depth of the same lobes. The percentage of mG^+^ thyroid cells was quantified on cryosections of thyroids (*n*=87) from *TgCreER^T2^;mTmG* and *TgCreER^T2^;Braf^CA/+^;mTmG* mice. Specimens were serial-sectioned transversely, encompassing the entire gland or at three standardized levels (equator and halfway to either lobe pole) in different experiments. Cell counting followed a systematic, unbiased protocol at ×20 magnification.

### Serum TSH and total T4 measurements

In euthanized animals, blood samples drawn from the heart were taken and, after clotting, centrifuged at 1800 rpm at 4°C for 10 min followed by collection of serum. Tetraiodothyronine (T4; identical to thyroxine) was analyzed using a total T4 ELISA kit (cat. no.: DNOV054, NovaTec, Germany). Absorbance was measured at 450 nm with the Wallac 1420 Victor2 microplate reader (Perkin Elmer, MA). The T4 concentration was calculated using MultiCalc 2000 software. Thyroid-stimulating hormone (TSH) was analyzed with the MILLIPLEX MAP Mouse Pituitary Magnetic Bead Panel (cat. no.: MPTMAG-49K, Merck Millipore, Germany). Fluorescence intensity was measured with the Luminex 200 analyzer (Luminex Corporation, Austin, TX) and data were analyzed using Luminex Xponent software v. 3.1.

### Magnetic resonance imaging (MRI)

Mouse MRI data were acquired by using a 7T horizontal bore preclinical MRI system with a 72-mm volume coil (Bruker BioSpin MRI GmbH, Germany; software: ParaVision 5.1), and a 4-channel array rat brain receiver coil for signal reception (RAPID Biomedical GmbH, Germany). Animals were imaged in supine position and kept under anesthesia using air, oxygen and isoflurane (2-3%, Isoba vet., Schering-Plough Animal-health, Denmark). A circulating warm-water and heating-pad system maintained the body temperature, and a pressure-sensitive pad monitored breathing (SA Instruments, Inc., NY). Anatomical MRI neck scans were acquired using a T2-weighted 2D RARE sequence with fat suppression (Repetition time: 4500 ms, Echo time: 35 ms, Number of averages: 16, Turbo factor: 6, Slice thickness/gap: 0.6/0.6 mm, Number of slices: 40, Pixel dimensions: 160×160 µm^2^, Field-of-view: 2.3×1.6 cm^2^). Following 1st and 2nd order automatic shimming, diffusion weighted images (DWIs) were acquired using the SE-EPI sequence (Repetition time: 3000 ms, Echo time: 21 ms, Number of averages: 3, Slice thickness/gap: 1.0/1.5 mm, Number of slices: 3, Pixel dimensions: 311×318 µm^2^, Field-of-view: 2.8×1.5 cm^2^: b-values: 0, 5, 10, 20, 35, 50, 75, 100, 200, 400, 600 and 800 s/mm^2^: bandwidth: 300 kHz). Apparent diffusion coefficient (ADC) maps were created online using mono-exponential fitting to data from all b-values.

### Whole-exome sequencing

For whole-exome sequencing (WES), genomic DNA was extracted with All prep DNA/RNA kit (Qiagen, Hilden, Germany) and sheared via sonication using a S220 focused-ultrasonicator (Covaris, Woburn, MA) from the thyroids and kidneys, used as reference tissue, of mutant mice (*n*=5). DNA libraries were constructed using the SureSelectXT Mouse All Exon kit (Agilent Technologies, Santa Clara, CA) and sequenced using Illumina's High Output Kit (150 cycles) on a NextSeq500 (Illumina, San Diego) with an average depth of 140× for tumor and 70× for normal (kidney) samples. Raw reads were mapped on the mouse reference genome GRCm38 using the BWA-MEM algorithm (Li and Durbin, 2009). The Picard command line tool set was used to remove PCR duplicates and local realignment was performed using the Genome Analysis Toolkit (GATK; McKenna et al., 2010). Somatic mutations were identified using MuTect2 (Cibulskis et al., 2013) with default settings using kidney as matched normal sample. Annotation of variants was carried out using ANNOVAR (Wang et al., 2010). The list of somatic mutations was filtered for a minimum coverage of ten reads, keeping only non-synonymous mutations that were supported by at least four reads. For validation by targeted sequencing, primers were designed using the Primer3 software (http://bioinfo.ut.ee/primer3/). PCR was performed according to standard methods and libraries were constructed using the Nextera XT DNA Library Preparation Kit (Illumina, San Diego, CA). Constructed libraries were sequenced on a NextSeq500 (Illumina). Raw reads were mapped on the mouse reference genome GRCm38 with BWA-MEM algorithm (Li and Durbin, 2009). The Picard set was used to remove PCR duplicates and local realignment around the insertion–deletion (indel) region was performed using the GATK (McKenna et al., 2010). Somatic mutations were identified using Varscan (Koboldt et al., 2009).

Deposited WES data are accessible at BioProject.

### Statistics

Statistical analyses were made using Prism 7 for Mac Os X version 7.0 (GraphPad Software, Inc.). Analyses included unpaired *t*-tests assuming Gaussian distribution, with graph error bars displaying mean±standard deviation (s.d.). *P*-values of <0.05 were considered statistically significant.

## Supplementary Material

Supplementary information
