# Supplementary material for: Tissue architecture delineates field cancerization in BRAFV600E-induced tumor development
Source: Dis Model Mech. 2021 Aug 11;15(2):dmm048887. doi: 10.1242/dmm.048887 (PMC8380047; doi:10.1242/dmm.048887)
Supplement: Supplementary information [file dmm-15-048887-s1.pdf]

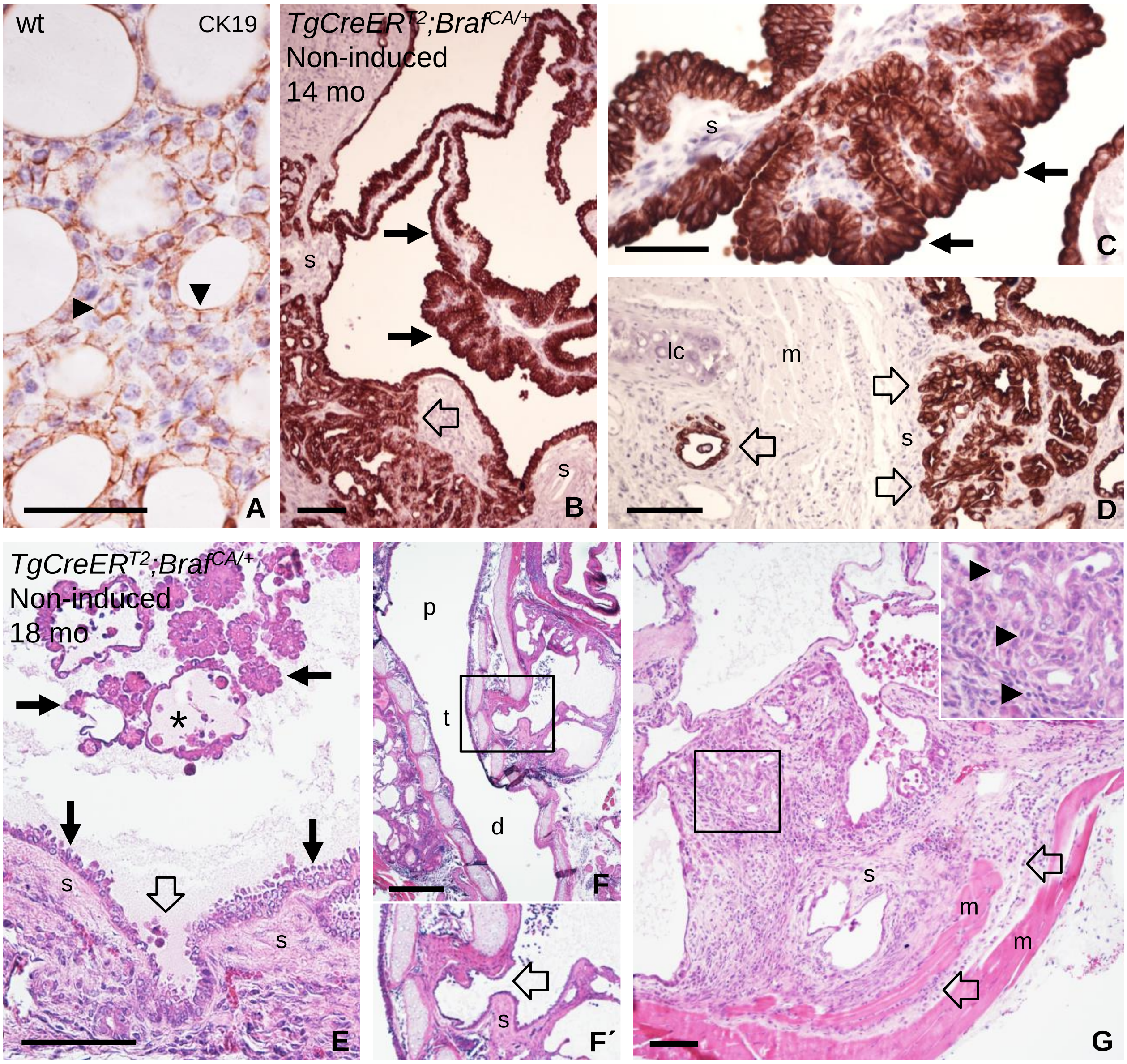

Fig. S1. Spontaneous *Braf*^{CA} activation generates CK19 positive papillary thyroid carcinoma (PTC) with signs of tumor invasiveness. Images obtained from 14 and 18 months (mo) old *TgCreER*^{T2}*;Braf*$^{CA/+}$ mice. CK19 immunohistochemistry (A-D). **A**, Weak CK19 expression in normal mouse thyroid. Arrowheads indicate preferential membranous localization of CK19 in follicular epithelial cells. **BC**, Strong expression of CK19 in tumor cells of a multilocular PTC. Arrows indicate tumorous epithelium lining enlarged follicular lumens and forming typical papillae. **D**, Stromal and extrathyroidal invasiveness of CK19^{+} thyroid tumor cells indicated by open arrows (also shown in B). B´ shows high power of boxed area in B. Note stromal infiltration of tumor cells (arrows). **E**, Cystic PTC with capsular invasion (open arrow). Note tumor cells display nuclear features typical of PTC and cell shape reminiscent of a hobnail-like phenotype. Asterisk indicates internal cavity of papillary formations projecting into the cyst.
F, Cystic tumor infiltrating the trachea (open arrow in F´ = high power of boxed area in F). **G**, Solid tumor originating from the wall of an enlarged follicle. Inset shows detail of boxed area with heterotypic tumor cells and tumor-infiltrating lymphocytes indicated by arrowheads. Open arrows indicate extrathyroidal tumor invasion. s, stroma; m, skeletal muscle, lc, laryngeal cartilage; t, trachea; p, proximal; d, distal. Scale bars: 500 (F), 100 (B, D, E, G) and 50 (A, C) µm.

Disease Models & Mechanisms • Supplementary information

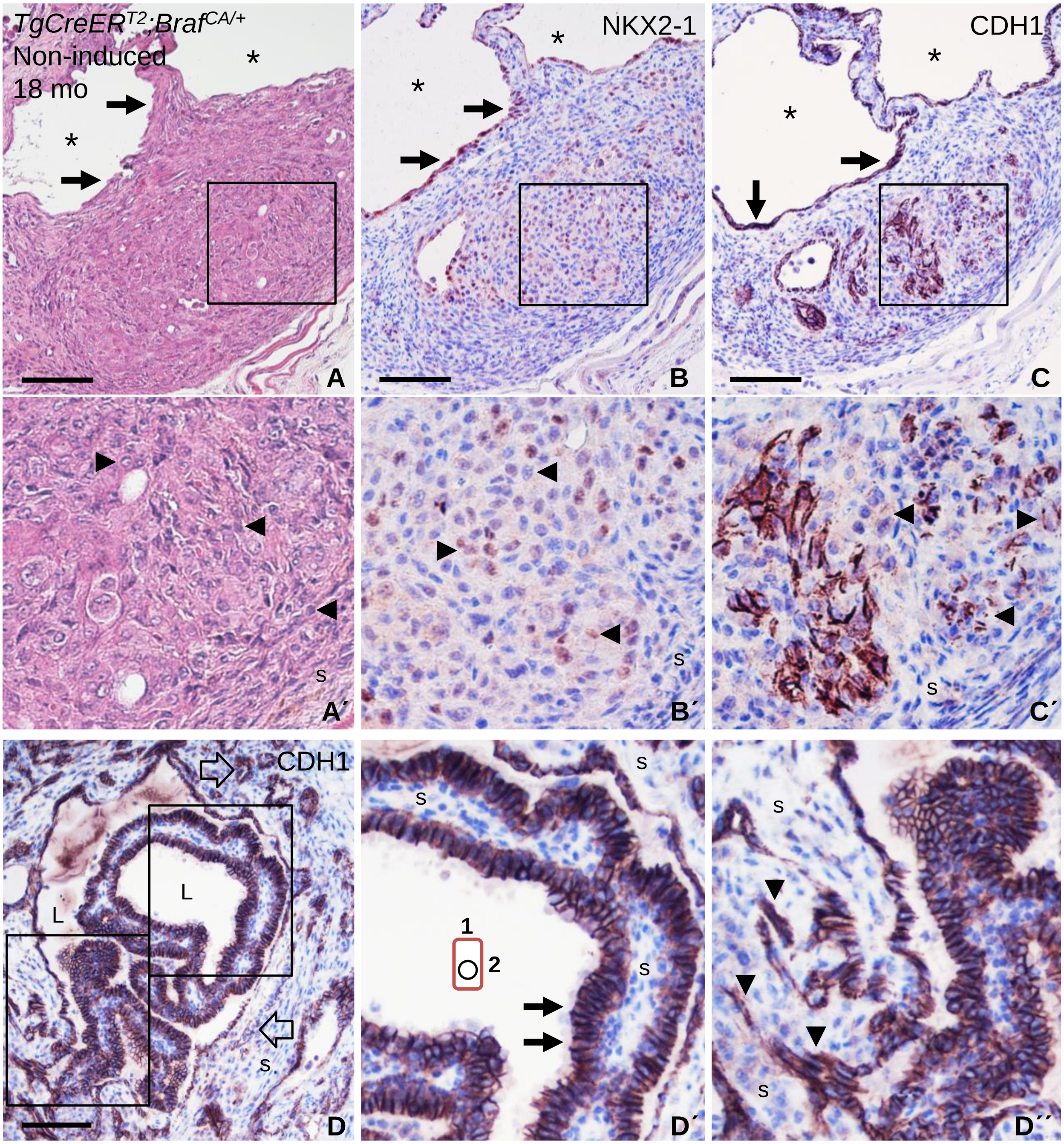

Fig. S2. Phenotype of tumor variants of papillary thyroid carcinoma (PTC) present in an 18 months old non-induced *TgCreER*^{T2}*;Braf*$^{CA/+}$ mouse. Figs. 1I-K and S1E-G derive from the same thyroid specimen. **A-C**, Solid variant of PTC adjacent to an enlarged, septate follicle (asterisks). Histology (A) and immunostaining for NKX2-1 (B) and E-cadherin (CDH1; C) on parallel sections. A´-C´ show high power of boxed areas. Arrows indicate flat epithelium strongly positive for both markers. Arrowheads show tumor cells with pleomorphic nuclei (A´), diminished expression of NKX2-1 (B´) and partial loss of CDH1 at cell contacts (C´). **D**, Tall-cell variant of PTC surrounded by abundant fibrous tissue intermixed with neoplastic follicles (large arrows). D´, D´´ highlight predominant cell shape with length/width ratio 2:1 or more (arrows and cartoon) in central tumor (D´) and signs of stromal invasion of CDH1+ tumor cells (D´´; arrowheads) . L, lumen of tumor; s, stroma. Scale bars (A-D): 100 µm.

Disease Models & Mechanisms • Supplementary information

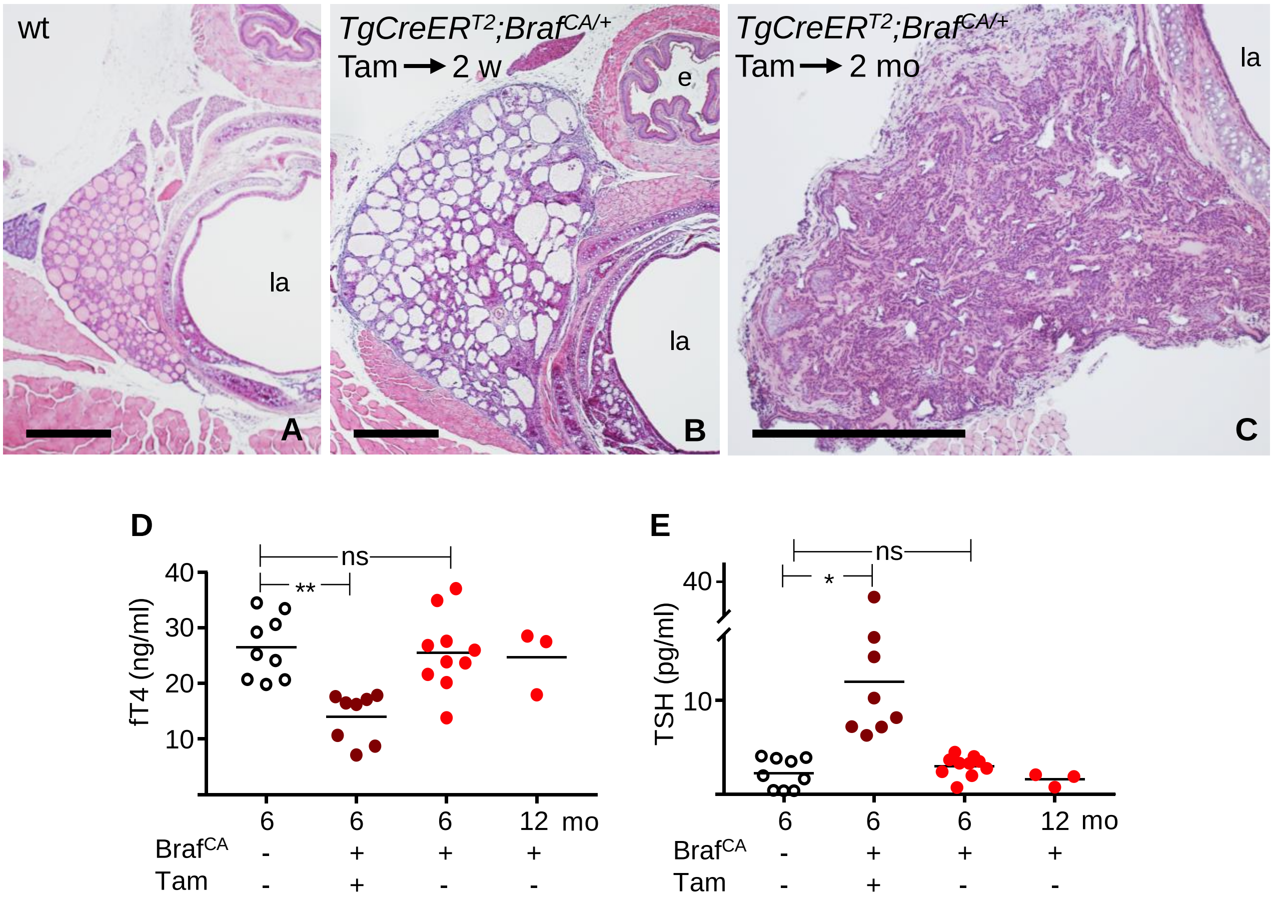

Fig. S3. Effects of induced *Braf*^{CA} activation in mouse thyroid. *TgCreER*^{T2}*;Braf*$^{CA/+}$ mice were injected or not with tamoxifen (tam; daily x3) after weaning. At times indicated, thyroids were excised and processed for microscopy of paraffin sections stained with hematoxylin-eosin, and peripheral blood was saved for measurements of thyroid stimulating hormone (TSH) and free thyroxin (fT4) levels. **A-C**, Cross section of right thyroid lobe in wildtype (wt) and Braf mutant mice 2 weeks (w) and 2 months (mo) after first tamoxifen injection. Note general morphological changes in mutant thyroids at both time points. la, larynx, cricoid level; e, esophagus. Scale bars: 500 µm. **DE**, Mean and individual circulating free T4 (fT4) and TSH levels in mutant mice with tamoxifen-induced or spontaneous (non-induced) Cre activation. Dots represent individual values. *, p=0.005; **, p=0.005. ns, not significant.

Disease Models & Mechanisms • Supplementary information

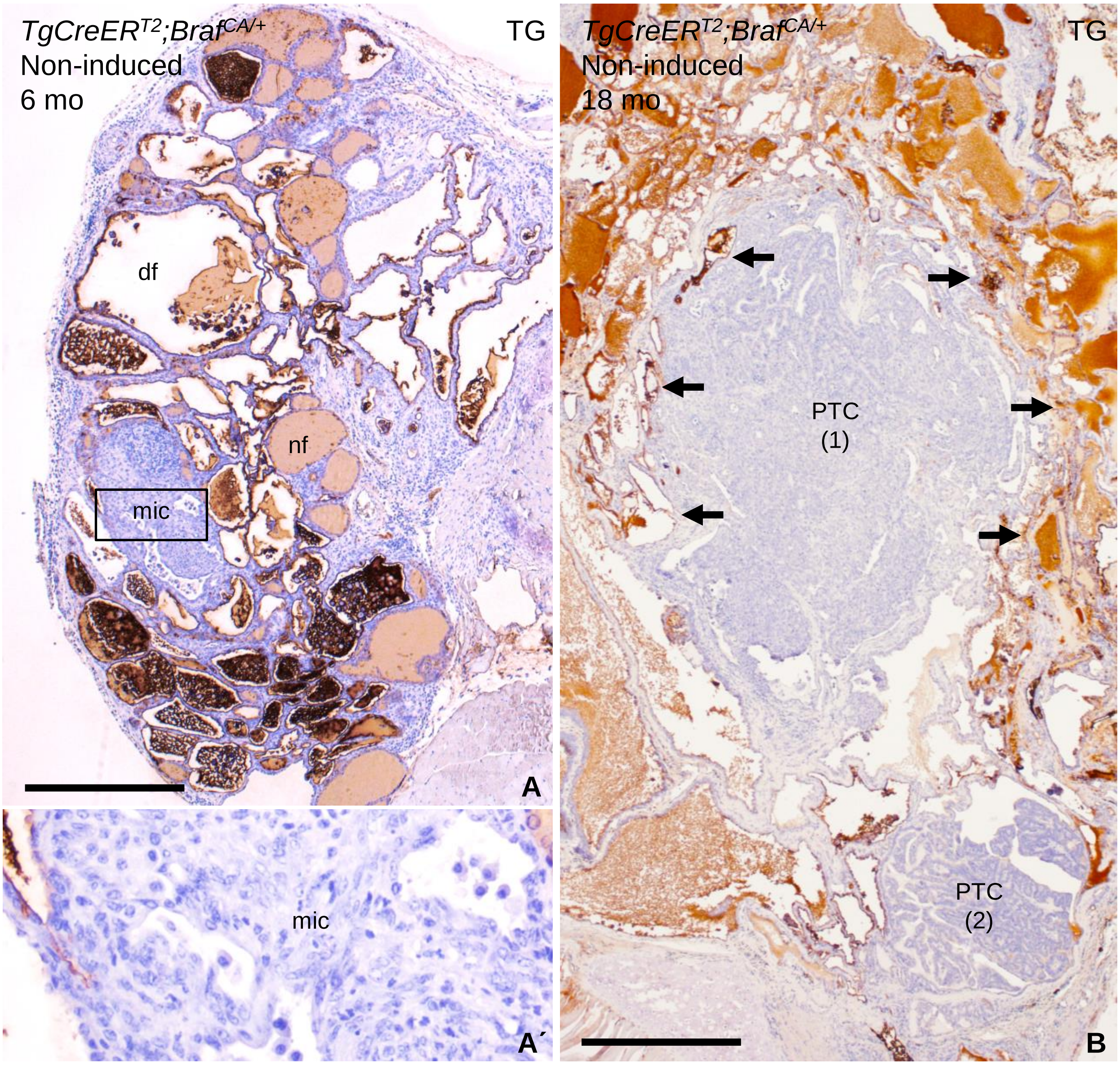

Fig. S4. Dedifferentiation – i.e. loss of thyroid function – of papillary thyroid carcinoma (PTC) cells in non-induced *TgCreER*^{T2}*;Braf*$^{CA/+}$ mice. Images with immunohistochemical staining of thyroglobulin (TG). **A**, Overview of right thyroid lobe in a 6 months mutant mouse. The tissue consists mainly of TG positive (TG+) follicles with homogeneous or disrupted colloid and a microcarcinoma (mic) entirely free of TG immunoreactivity. A´ shows high power of boxed area. nf, normal follicle; df, dilated follicle. **B**, Advanced tumor stage in an 18 month old mutant. Image from a parallel section to that shown in Fig. 1I. Only the two carcinomas (1 and 2) are devoid of TG; TG+ follicles are present in the marginal zone of tumors (arrows). Scale bars (A, B): 500 µm.

Disease Models & Mechanisms • Supplementary information

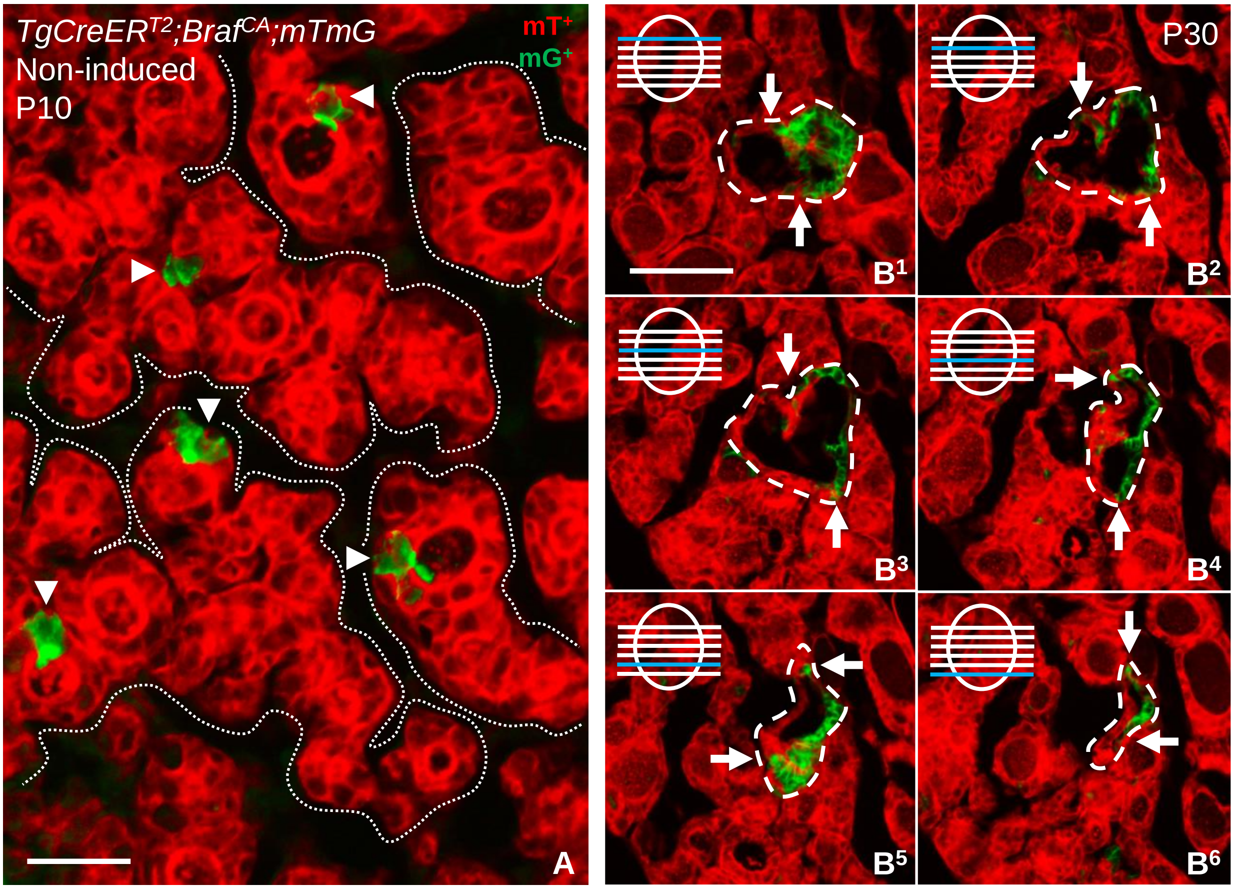

Fig. S5. Tracing spontaneous *Braf^{CA}* activation and clonal development of Braf mutant cells in the postnatal thyroid gland. *TgCreER^{T2}; Braf$^{CA/+}$* mice were recombined with the double fluorescent *mTmG* reporter to generate *TgCreER^{T2}; Braf$^{CA/+}$;mTmG* mice. **A**, Incidence of *mTmG* activation as revealed at postnatal day 10 (P10). Arrowheads indicate single mG^{+} follicle cells present in premature follicles. Branching network of parenchyma is outlined (dotted lines). **B**, Serial sections (B^{1}-B^{6}) of an enlarged, dual labeled (compound mT^{+}/mG^{+}) follicle with an irregular, abnormal shape (encircled). Section levels with reference to the enlarged follicle are indicated in upper, left corner; B^{3} is identical to Fig. 4H. Note the follicle consists of two differentially labeled, contiguous epithelial domains of equal size. Arrows indicate transition sites of mT^{+} and mG^{+} clones. Scale bars: 100 (B) and 25 (A) µm.

Disease Models & Mechanisms • Supplementary information

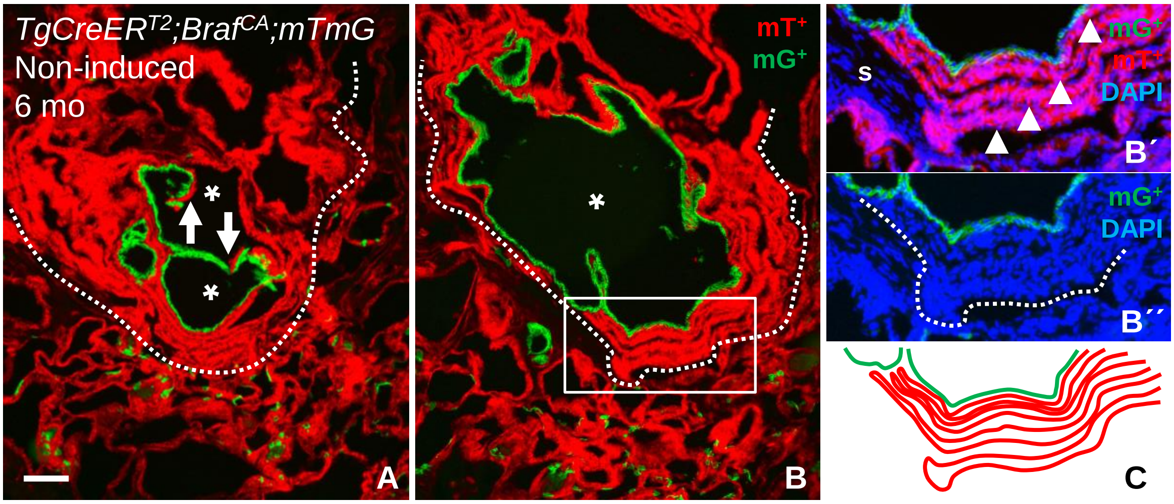

Fig. S6. Clonal selection of growth in papillary thyroid carcinogenesis following stochastic *Braf*^{CA} activation. Thyroid tissue from a 6 month (mo) old *TgCreER*^{T2}*;Braf*$^{CA/+}$*;mTmG* mouse. **A, B**, Serial images of a dual labeled tumor (encircled) indicating predominant papillary growth of a mT^+ clone entrapping a mG^+ clone that essentially faces the enlarged lumen (asterisks). Arrows indicate clonal transition sites of the originating oligoclonal epithelium. B´, B´´ show boxed area in B with additional DAPI staining illustrating multilayered folding (arrowheads) of the mT^+ neoplastic epithelium. **C**, Cartoon of B´ for clarity. s, stromal tissue surrounding tumor. Scale bar: 100 µm.

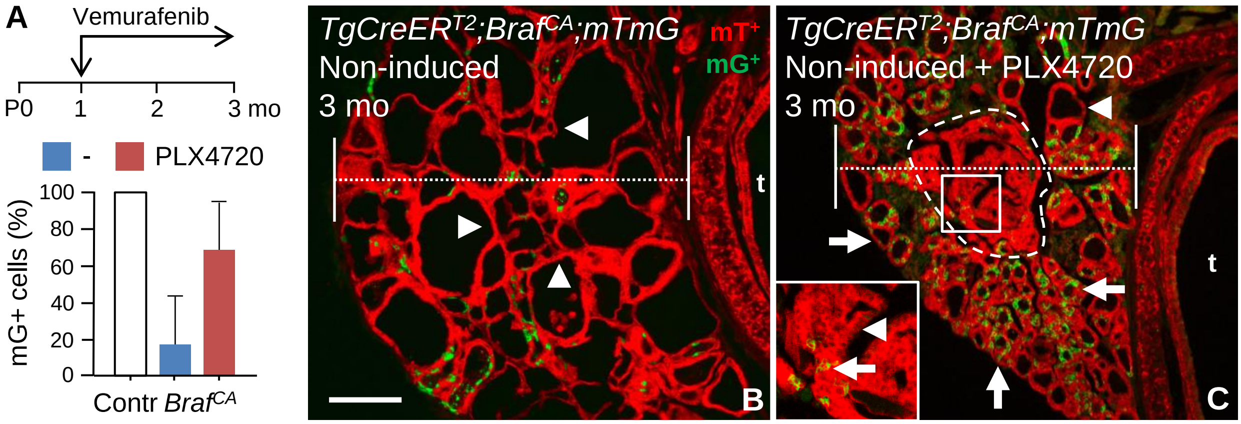

Fig. S7. Resistance of tumorigenic clones to $Braf^{V600E}$ kinase inhibition. *TgCreER^{T2};Braf$^{CA/+}$;mTmG* mice were fed PLX4720 (417 ppm) from weaning and sacrificed at 3 months of age. Serial sections were counted for mG^+ thyroid cells and compared to that of age-matched *TgCreER^{T2};mTmG* mice. **A**, Maintained reporter gene activation in drug-treated mutants. Mean±sd (n): controls (6), untreated mutants (6), tamoxifen-treated mutants (6); three section levels/gland. **B, C**, Normalization of lobe size (indicated by brackets), follicle size and mG^+ labeling of cells by vemurafenib. Note preserved mT+ labeling of microcarcinoma cells (encircled). Inset shows boxed area in C. Arrows, G^+ cells; arrowheads, mT^+ cells; t, trachea. Scale bar: 200 µm.

Table S1. Quality data from whole exome sequencing of thyroid carcinomas generated by spontaneous activation of *Braf*^{V600E} in *TgCreER*^{T2}*;Braf*$^{CA/+}$ mice.

Mouse Id	Sample[1]	Total number of reads	Mapped number of reads	% of mapped reads	Mean coverage
3182	Tumor	60027679	59294617	98.78	185.36
	Normal	28873205	28555696	98.91	90.0824
4187	Tumor	57690485	57690485	98.96	173.888
	Normal	43839295	43360247	98.91	135.016
4655	Tumor	85800968	84806007	98.85	260.843
	Normal	29903775	29544091	98.8	92.7111
4743	Tumor	71108114	69976003	98.41	209.029
	Normal	23167811	22835561	98.57	69.0573
4747	Tumor	73639412	72888030	98.98	223.971
	Normal	18999407	18809921	99.01	58.0215

[1]Normal refers to constitutional control tissues (kidney) obtained from the same mice

Table S2. Gene expression changes associated with dedifferentiation of Braf mutant thyroid cells after spontaneous and induced *Braf*^{CA} activation in *TgCreER*^{T2}*;Braf*$^{CA/+}$ mice.

	qPCR[1]				
Tam[2]	*Pax8*	*Tg*	*Slc5a5/Nis*	*Tpo*	*Tshr*
-	0,90 ± 0,19	0,57 ± 0,20	0,16 ± 0,03	0,45 ± 0,06	0,63 ± 0,11
+	0,25 ± 0,02	0,03 ± 0,01	0,15 ± 0,06	0,13 ± 0,04	0,19 ± 0,03

[1]Analysis of thyroid tissue samples obtained from 5 weeks + 10 days (± post-injection) old mice (n=3/group)
[2]Tamoxifen; i.p. injections daily x3 after weaning
[3]Mean ± sd; expression relative to transcript levels in control (*TgCreER*^{T2}) mice set to “1”

Disease Models & Mechanisms • Supplementary information

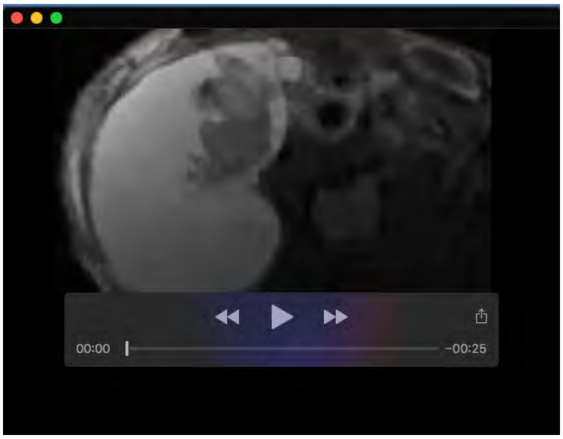

Movie 1. Magnetic resonance imaging (MRI) of multicentric papillary thyroid carcinomas. Scrolling stack of images based on transverse MRI encompassing the entire thyroid gland and surrounding neck tissues in a 14 month old *TgCreER*^{T2}*;Braf*$^{CA/+}$ mouse subjected to spontaneous (non-induced) *Braf*^{CA} activation. For technical data and keys to image details, see Materials & Methods and accompanying Figs. 1G and H. Image resolution is inherited to technical constraints.
